# Mathematical Modeling Highlights the Complex Role of AKT in TRAIL-Induced Apoptosis of Colorectal Carcinoma Cells

**DOI:** 10.1016/j.isci.2019.01.015

**Published:** 2019-01-14

**Authors:** Matthew W. Anderson, Joanna J. Moss, Robert Szalai, Jon D. Lane

**Affiliations:** 1 Centre for Biomedical Modelling and Analysis, Living Systems Institute, University of Exeter, Stocker Road, Exeter EX4 4QD, UK; 2 Cell Biology Laboratories, School of Biochemistry, University of Bristol, Medical Sciences Building, University Walk, Bristol BS8 1TD, UK; 3 Department of Engineering Mathematics, University of Bristol, Merchant Venturers Building, Woodland Road, Bristol BS8 1UB, UK

**Keywords:** Cancer, Cell Biology, *In Silico* Biology

## Abstract

Protein kinase B/AKT is a highly connected protein involved in a range of signaling pathways. Although it is known to regulate several proteins in the apoptotic pathway, its system-level effects remain poorly understood. We investigated the dynamic interactions between AKT and key apoptotic proteins and constructed a deterministic ordinary differential equation protein interaction model of extrinsic apoptosis. Incorporating AKT and its indirect inhibitor, phosphatase and tensin homolog (PTEN), this was used to generate predictions of system dynamics. Using eigen analysis, we identified AKT and cytochrome *c* as the protein species most sensitive to perturbations. Cell death assays in Type II HCT116 colorectal carcinoma cells revealed a tendency toward Type I cell death behavior in the *XIAP*^*−/−*^ background, with cells displaying accelerated TRAIL-induced apoptosis. Finally, AKT inhibition experiments implicated AKT and not PTEN in influencing apoptotic proteins during early phases of TRAIL-induced apoptosis.

## Highlights

• TRAIL-induced apoptosis model describes AKT protein interaction dynamics
• AKT and cytochrome *c* identified as the proteins most sensitive to perturbations
• HCT116 cells shift from Type II to Type I cell death behavior in *XIAP*^*−/−*^ background
• AKT and not PTEN influences early phases of TRAIL-induced apoptosis

## Graphical Abstract

**Figure:**
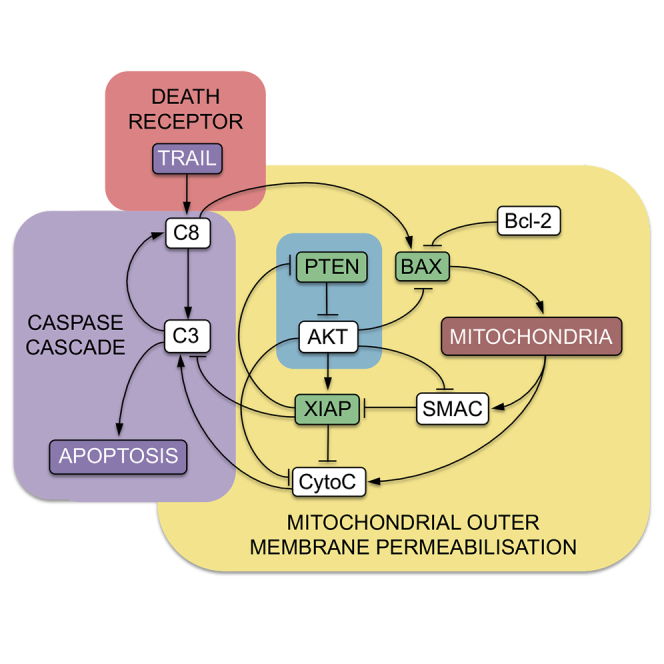

## Introduction

Apoptosis is executed by caspases that are activated via intrinsic and extrinsic signaling pathways (Scaffidi et al., 1998). The intrinsic pathway is initiated by DNA damage, substrate detachment, or growth factor withdrawal and involves mitochondrial outer membrane permeabilization (MOMP), and the release of cytochrome *c* (Fulda and Debatin, 2006). The extrinsic pathway is induced by ligand binding to plasma membrane receptors of the tumor necrosis factor superfamily, and the downstream molecular cascade that is triggered is believed to be genetically determined. This pathway can trigger two types of cell death signaling. First, Type I cells such as lymphocytes undergo mitochondria-independent cell death, relying solely on a receptor or ligand-instigated caspase cascade (Barnhart et al., 2003, Scaffidi et al., 1998). In Type II cells, however, amplification through MOMP and cytochrome *c* release is necessary (Scaffidi et al., 1998). Understanding how specific cells coordinate apoptotic responses contributes to our appreciation of cell death dynamics in disease.

AKT (protein kinase B) is a promiscuous serine/threonine-specific protein kinase that influences protein synthesis (Wu, 2013), proliferation (Dong et al., 2015), glucose metabolism (Kornfeld et al., 2013), synaptic signaling (Liu et al., 2015), autophagy (Heras-Sandoval et al., 2014, Wang et al., 2012), and nuclear factor-κB signaling (Davoudi et al., 2014). Several studies have also revealed a pivotal role for AKT in apoptosis. AKT inhibits apoptosis via inhibitory phosphorylation of the pro-apoptotic BCL-2 homology domain 3 (BH3-only) protein BAD (del Peso et al., 1997), triggering a cascade of inhibitory reactions impinging on pro-apoptotic BAX (AKT ┤ BAD ┤ BCL-2 ┤ BAX; ┤ denoting inhibition). The BCL-2-BAX and BAD-BCL-2 interactions are direct binding associations dependent on their respective BCL-2 homology (BH) domains, whereas AKT inactivates BAD through phosphorylation at Ser^136^ leading to AKT sequestration by 14-3-3 proteins (del Peso et al., 1997). AKT also phosphorylates BAX at Ser^184^, preventing the conformational changes in BAX needed for oligomerization and pore-forming capabilities during MOMP (Wang et al., 2010). Downstream of MOMP, AKT phosphorylates procaspase-9 at Ser^196^, preventing its processing and activation (Cardone et al., 1998). It also phosphorylates the X-linked inhibitor of apoptosis protein (XIAP) (Deveraux and Reed, 1999), an E3 enzyme that ubiquitylates caspases 9, 3, and 7, targeting them for proteasomal degradation. XIAP also regulates its own stability through autoubiquitylation (Nakatani et al., 2013), a process that is blocked by AKT-mediated Ser^87^ phosphorylation (Dan et al., 2004). Robust cell death initiation requires XIAP inhibition via SMAC (second mitochondria-derived activator of caspases) that is released during MOMP and binds to the tetrapeptide IAP-binding motif of XIAP (Scott et al., 2005). AKT phosphorylates SMAC at Ser^67^ to increase its binding to XIAP, conferring resistance to apoptosis (Jeong et al., 2015).

Any systems-level study of the role of AKT during apoptosis must consider PTEN (phosphatase and tensin homolog). PTEN acts as a positive regulator of apoptosis by antagonizing AKT activation (Baehrecke, 2005); however, it is also downregulated via XIAP-mediated ubiquitylation and degradation (Van Themsche et al., 2009). In this study, we have constructed a deterministic model of apoptosis incorporating the interactions between AKT, PTEN, and the apoptotic machinery. System dynamics predictions generated using this model describe how individual protein species as well as the apoptotic system as a whole are affected in different genetic backgrounds. This model accurately predicts protein dynamics for three of four HCT116 cell lines (wild-type; *BAX*^*−/−*^; *XIAP*^*−/−*^), and further investigations uncover the possible mechanisms behind the dynamics of the fourth (*PTEN*^*−/−*^). Subsequent laboratory studies show that AKT regulation of apoptosis is significantly stronger during TRAIL-mediated extrinsic apoptosis than in TRAIL-independent apoptosis in these cells, and that its effects are more prominent at early points in the apoptotic response.

## Results

### The AKT Apoptosis Model Predicts Protein Dynamics in Different Genetic Backgrounds

**Figure 1:**
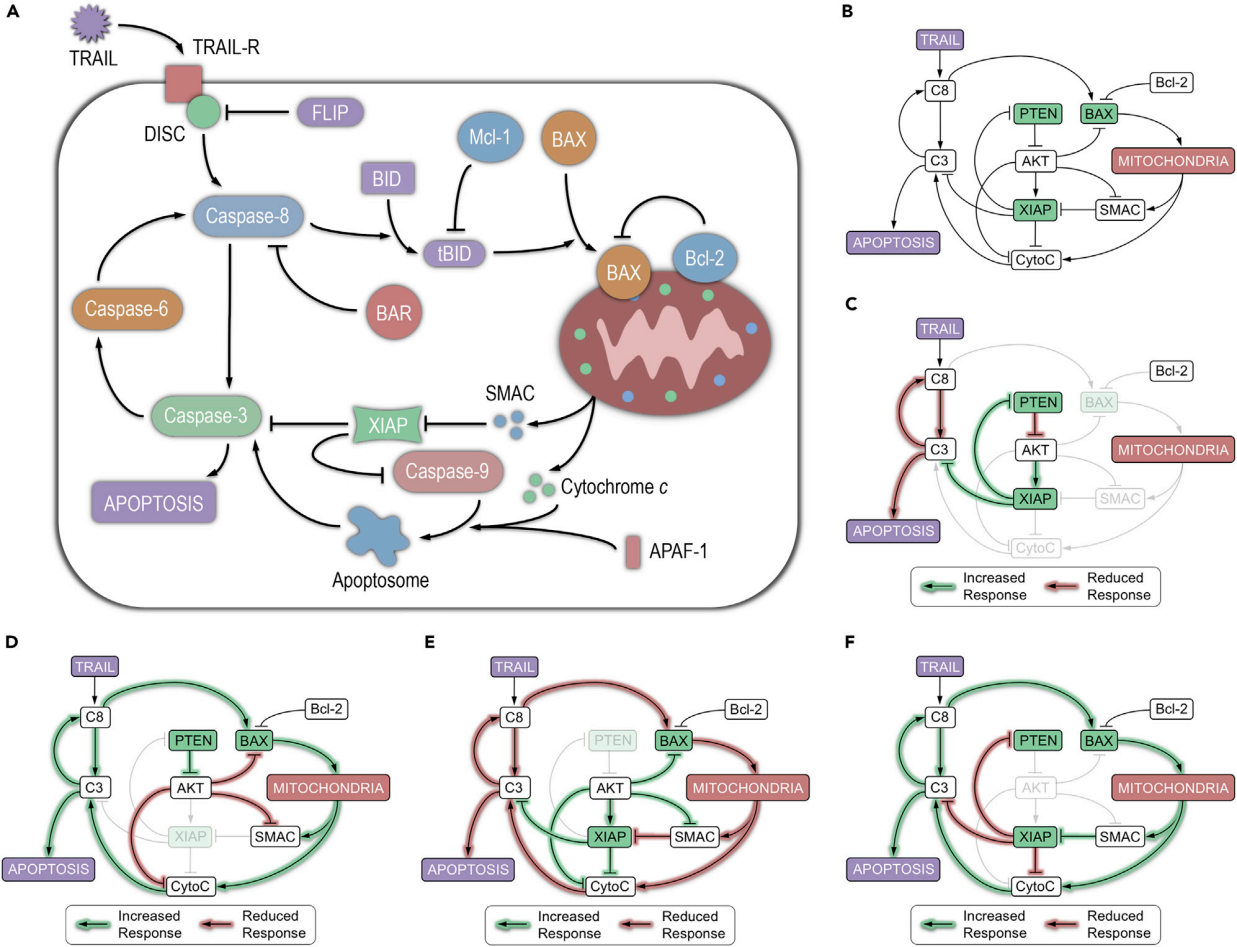
The AKT Apoptosis Model Predicts Protein Dynamics in Different Genetic Backgrounds (A) Extrinsic apoptosis signaling pathway. (B) The AKT apoptosis model (AKTM). Protein species shown in green are those for which double knockout HCT116 cell lines are used in this study. TRAIL, TNF-related apoptosis-inducing ligand; C8, active caspase-8; BAX, Bcl-2-associated X protein; Bcl-2, B-cell lymphoma 2; SMAC, second mitochondria-derived activator of caspases; CytoC, cytochrome *c*; XIAP, X-linked inhibitor of apoptosis protein; AKT, protein kinase B; PTEN, phosphatase and tensin homolog; C3, active caspase-3. (C) Predicting the effects of BAX removal on the AKTM. Green lines signify a predicted increased response, and red lines signify a reduced response. (D) Predicted effects of XIAP removal on the AKTM. (E) Predicted effects of PTEN removal on the AKTM. (F) Predicted effects of AKT removal on the AKTM.

**Figure 2:**
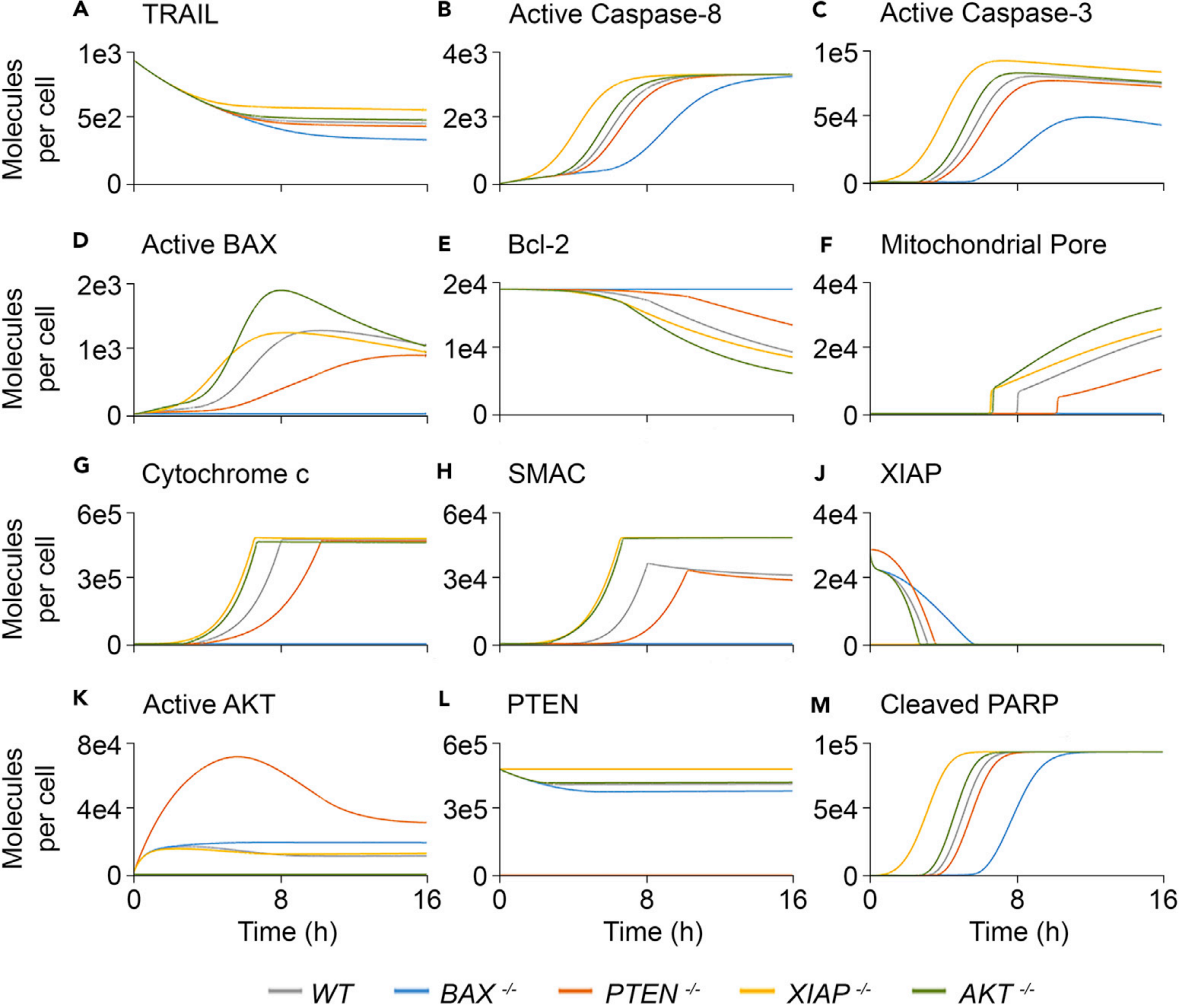
AKTM Simulation Results (A–H and J–M) Simulated concentrations of the following protein species in the HCT116 wild-type, *BAX*^*−/−*^, *PTEN*^*−/−*^, *XIAP*^*−/−*^, and *AKT*^*−/−*^ cell lines for a 16-h period following exposure to TRAIL and cycloheximide: (A) TRAIL, (B) active caspase-8, (C) active caspase-3, (D) active BAX, (E) Bcl-2, (F) mitochondrial pore, (G) cytochrome *c*, (H) SMAC, (J) XIAP, (K) active AKT, (L) PTEN, and (M) cleaved PARP.

Using a simplified model of extrinsic apoptosis as a starting point (Figure 1A), we constructed the AKT apoptosis model (AKTM) (Figure 1B), a deterministic, single-cell model wherein protein-protein interactions are described by ordinary differential equations and outputs report temporally variable protein concentrations (Methods). Figures 1C–1F are schematics depicting hypothesized effects of removing the influential proteins BAX, PTEN, XIAP, and AKT from the system, and the outcomes of AKTM simulations in these hypothetical scenarios are shown in Figure 2.

Simulated concentrations of active caspase-8 in all scenarios settled at or close to the same steady state by the end of the simulation. The first condition to reach steady state was *XIAP*^*−/−*^ (∼8 h), followed by (1) *AKT*^*−/−*^ (∼10.8 h), (2) wild-type (∼11 h), (3) *PTEN*^*−/−*^ (∼11.3 h), and (4) *BAX*^*−/−*^, which had not reached steady state by 16 h (Figure 2B). The active caspase-3 response curves did not settle at the same steady state, but instead reached peak concentrations at different magnitudes and times—the higher the peak, the earlier it occurred—in the following order: (1) *XIAP*^*−/−*^ (∼7 h), (2) *AKT*^*−/−*^ (8 h), (3) wild-type (9 h), (4) *PTEN*^*−/−*^ (10 h), and (5) *BAX*^*−/−*^ (12 h) (Figure 2C). Simulated concentrations of active caspase-3 were similar in *AKT*^*−/−*^ and *PTEN*^*−/−*^ simulations, whereas they were ∼15% higher and ∼40% lower, respectively, in wild-type and *XIAP*^*−/−*^ states (Figure 2C).

Active BAX concentration peaked at ∼10 h in the wild-type simulation and at ∼8 h in *XIAP*^*−/−*^, but required ∼16 h for *PTEN*^*−/−*^ (Figure 2D). Peak active BAX concentration was similar in the wild-type and *XIAP*^*−/−*^ simulations, but almost 30% lower in the absence of PTEN. The active BAX peak in the *AKT*^*−/−*^ simulation was approximately 50% higher than in wild-type, highlighting the AKT-imposed inhibition of BAX. Cytochrome *c* (Figure 2G) and SMAC (Figure 2H), the “products” of MOMP, are not released from the mitochondria in the absence of BAX and therefore never become cytosolic in the *BAX*^*−/−*^ simulation. Compared with wild-type, their release was slower in the *PTEN*^*−/−*^ state, but faster in *AKT*^*−/−*^ and *XIAP*^*−/−*^ scenarios. Cytoplasmic cytochrome *c* concentration settled at roughly the same steady state in these conditions (Figure 2G), but cytosolic SMAC concentration began to decay in the wild-type and *PTEN*^*−/−*^ simulations (Figure 2H).

In the *PTEN*^*−/−*^ simulation, XIAP concentration did not begin to decay as quickly as in the other states, remaining the highest for the first 2–3 h until its decay speed increased (Figure 2J). Henceforth, XIAP concentration became greatest in the *BAX*^*−/−*^ state, eventually fully depleting in all simulations by the 6-h mark (Figure 2J). The *PTEN*^*−/−*^ simulation also showed a comparatively large peak in active AKT concentration, evidence of the modeled AKT inhibition by PTEN (Figure 2K).

The concentration-response curves for the aforementioned proteins are useful for understanding the underlying mechanisms governing system dynamics, but the overall apoptotic response is reported in the response curves for cleaved poly(ADP-ribose) polymerase (PARP) (Figure 2M). These presented the same sigmoid shape in each scenario, the only difference being the timing of the increase phase, which was initiated after 1 h in *XIAP*^*−/−*^, 2.6 h in *AKT*^*−/−*^, 3.2 h in wild-type, 3.8 h in *PTEN*^*−/−*^, and 5.7 h in *BAX*^*−/−*^. In all simulations, the increase phase lasted for ∼4.5 h, such that steady state was reached at 5.3 h in *XIAP*^*−/−*^, 7.1 h in *AKT*^*−/−*^, 7.7 h in wild-type, 8.3 h in *PTEN*^*−/−*^, and 10.2 h in *BAX*^*−/−*^ (Figure 2M).

### Eigen Analysis Reveals Perturbation Sensitivity of AKT and Cytochrome *c*

**Figure 3:**
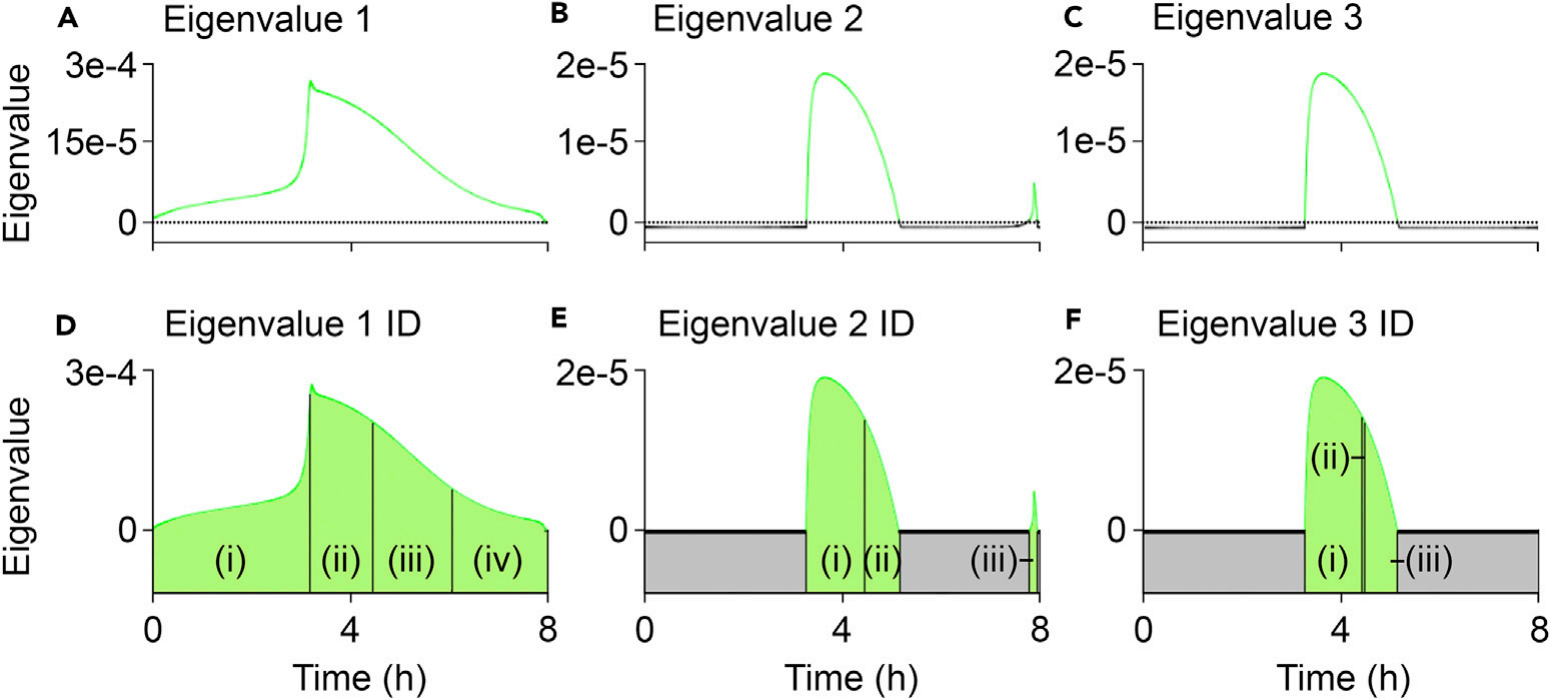
Eigen Analysis Reveals Perturbation Sensitivity of AKT and Cytochrome *c* (A–C) Eigen analysis of the three positive eigenvalues present in the wild-type system: (A) eigenvalue 1 (most positive), (B) eigenvalue 2, and (C) eigenvalue 3 (least positive). (D) The temporally variable protein species most responsible for eigenvalue 1 dynamics: (i) active AKT, (ii) PARP, (iii) cytosolic cytochrome *c*, and (iv) mitochondrial cytochrome *c*. (E) Eigenvalue 2 dynamics: (i) cleaved PARP, (ii) mitochondrial cytochrome *c*, and (iii) cytosolic cytochrome *c*. (F) Eigenvalue 3 dynamics: (i) cytosolic cytochrome *c*, (ii) cleaved PARP, and (iii) PARP.

Eigen analysis was performed on the wild-type variant of the model, and of the 41 eigenvalues computed only three were positive at any point during the simulation. Positive eigenvalues indicate instability, and their corresponding eigenvectors represent the protein species whose relative concentrations have changed (Methods). The three positive eigenvalues are shown in Figures 3A–3C, along with their corresponding most sensitive temporally variable protein species in Figures 3D–3F.

Active AKT was identified as the protein species most sensitive to perturbations (Figure 3D, i), as calculated from eigenvalue 1 (Figure 3A) from the start of the simulation to a few minutes after the 3-h mark, the point at which AKT concentration peaked (Figure 2K). Subsequently, PARP governed model dynamics until 4.4 h (Figure 3D, ii), when cleaved PARP concentration reached a steady increase (Figure 2M). Cytosolic cytochrome *c* then became dominant, corresponding to the middle of its exponential phase in the simulation (Figure 2G). Finally, mitochondrial cytochrome *c* assumed responsibility at the 6-h mark (Figure 3D, iii), when the rate of increase in cytosolic cytochrome *c* began to become stable (Figure 2G). This was also the point at which the rate of increase in cleaved PARP concentration began to slow (Figure 2M). Mitochondrial cytochrome *c* maintained dominance of eigenvalue 1 dynamics until 8 h (Figure 3D, iv), when complete cell death had been reached (signified by the concentration of cleaved PARP reaching its maximum steady state [Figure 2M]).

Eigenvalue 2 only became positive after 3.3 h when cleaved PARP was dominating its dynamics (Figure 3E, i). This continued until mitochondrial cytochrome *c* took over at 4.4 h (Figure 3E, ii) once the increase in cleaved PARP had become steady (Figure 2M) and the increase in the rate of release of mitochondrial cytochrome *c* was in its exponential phase (Figure 2G). The eigenvalue became negative at 5.1 h and could therefore not be regarded as unstable after this period. It did, however, become positive again for 0.2 h immediately before the 8-h mark, at which point cytosolic cytochrome *c* governed its dynamics (Figure 3E, iii). Taken together with eigenvalue 1, the period from 4.4–8 h is dominated by cytochrome *c* release dynamics as it is largely the mitochondrial and cytosolic cytochrome *c* species that control the dynamics of eigenvalues 1 and 2 during this period.

Eigenvalue 3 also only became positive after 3.3 h, at which point cytosolic cytochrome *c* was dominating its dynamics (Figure 3F, i). At the 4.4-h mark, cleaved PARP briefly (∼2 min) took over (Figure 3F, ii), before PARP assumed greater responsibility for eigenvalue 3 dynamics until 5.1 h, while it was still positive (Figures 3F, iii). The eigenvalue was then negative for the remainder of the simulation, and there are no other positive eigenvalues to identify. In addition to the sensitivity to perturbations of cytochrome *c* revealed by eigenvalues 1 and 2, eigenvalue 3 highlights the sensitivity of PARP, but only until MOMP transition initiated (i.e., the point of no return).

### Cell Death Assays Validate AKTM Predictions

**Figure 4:**
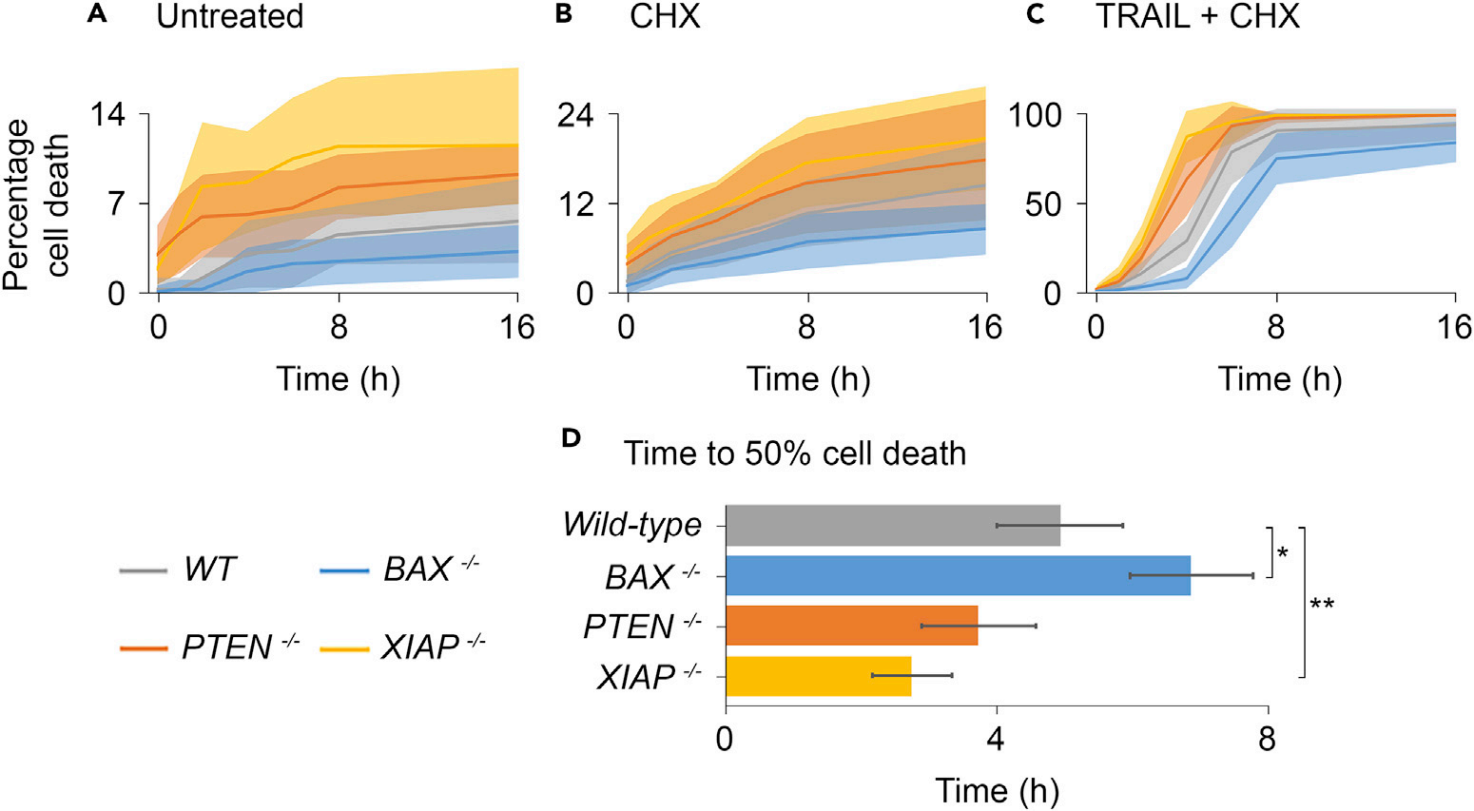
Cell Death Assays Validate AKTM Predictions (A) Percentage cell death in untreated (control) HCT116 cells. (B) Percentage cell death in HCT116 cells treated with 2.5 μg/mL cycloheximide. (C) Percentage cell death in HCT116 cells treated with 50 ng/mL TRAIL and 2.5 μg/mL cycloheximide. (D) Time to 50% cell death in HCT116 cells treated with 50 ng/mL TRAIL and 2.5 μg/mL cycloheximide. *BAX*^*−/−*^ cells reached 50% cell death significantly later than in wild-type (p < 0.05), *PTEN*^*−/−*^ cells did not significantly differ from wild-type, and *XIAP*^*−/−*^ cells reached 50% cell death significantly earlier (p < 0.01). Data in (A–D) represent means ± SD from three independent biological repeats. SDs are represented by shaded areas in (A–C) and by error bars in (D). * in (D) indicates a mean significantly different from that of the wild-type cell line with p ≤ 0.05 (Welch two-sample t test); ** signifies p ≤ 0.01. Detailed statistical results are displayed in Table S5.

We next tested the accuracy of our model *in vitro* using *BAX*^*−/−*^, *PTEN*^*−/−*^, and *XIAP*^*−/−*^ HCT116 cell lines. Figure 4 shows cell death assay results represented as percentage cell death for control/untreated (Figures 4A), 2.5 μg/mL cycloheximide (CHX)-treated (Figure 4B), and 50 ng/mL TRAIL + 2.5 μg/mL CHX-treated cells (Figure 4C). As expected, TRAIL + CHX (Figure 4C) caused the highest cell death: by the end of the 16-h imaging period all cells were dead in the *PTEN*^*−/−*^ and *XIAP*^*−/−*^ lines, whereas cell death in the *BAX*^*−/−*^ (83.4% ± 2.8%) and wild-type (95.1% ± 2.1%) lines was marginally delayed. Time taken to achieve 50% cell death was also recorded for these treatments (Figure 4D), demonstrating significantly delayed apoptosis in the *BAX*^*−/−*^line and significantly advanced apoptosis in the *XIAP*^*−/−*^ line. There was no significant difference between the *PTEN*^*−/−*^ and wild-type lines.

The most obvious (although minor) discrepancy between simulation and experimental data was seen in the *PTEN*^*−/−*^ line. Here, the model predicted a slight delay in the onset of apoptosis, whereas the data reported an advanced apoptotic onset, despite similar time to 50% cell death recordings (Figure 4D). This may be due to inaccurate model parameterization; however, we chose to investigate this further through protein quantification.

### Absence of BAX Expression in *PTEN*^*−/−*^ HCT116 Cells

**Figure 5:**
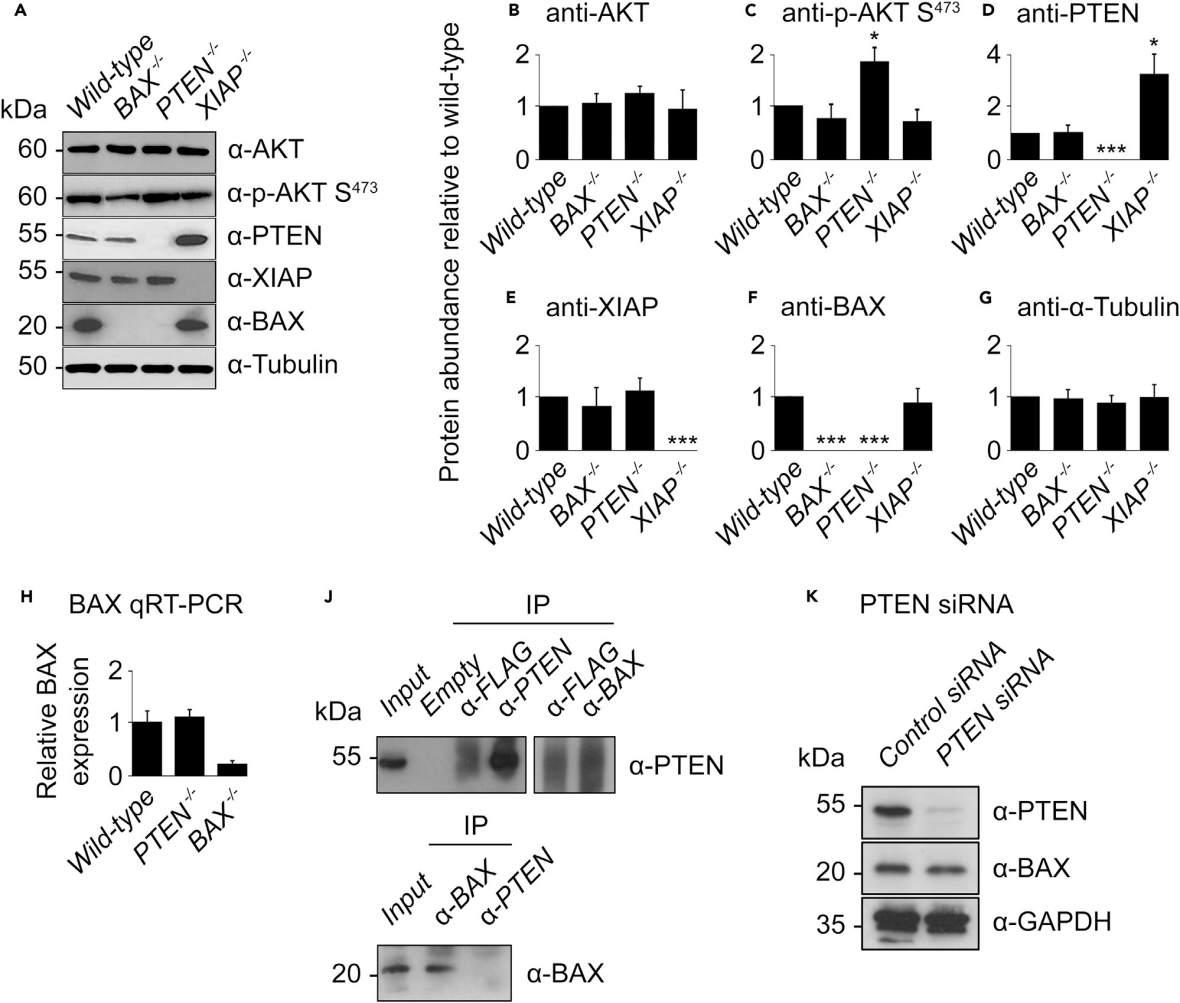
Absence of BAX Expression in *PTEN*^*−/−*^ HCT116 Cells (A–G) (A) Immunoblotting via western blot of wild-type, *BAX*^*−/−*^, *PTEN*^*−/−*^, and *XIAP*^*−/−*^ HCT116 cell lines for the following proteins: AKT, p-AKT (Ser^473^), PTEN, XIAP, BAX, and α-tubulin as a loading control. Densitometry quantitation of (B) AKT, (C) p-AKT Ser^473^, (D) PTEN, (E) XIAP, (F) BAX, and (G) tubulin. (H) qRT-PCR of BAX mRNA levels in wild-type, *PTEN*^*−/−*^, and *BAX*^*−/−*^ cells. (J) Immunoblot of immunoprecipitated endogenous BAX and PTEN from wild-type HCT116 cells; FLAG antibody used as a negative control, and 5% of protein lysate used as control for protein expression. (K) siRNA-mediated silencing of PTEN in wild-type HCT116 cells. Data in (B–G) represent normalized means (relative to wild-type = 1) from three independent biological repeats ± SD. * indicates a mean significantly different from that of the wild-type cell line with p ≤ 0.05 (Welch two-sample t test); *** signifies p ≤ 0.001. Detailed statistical results are displayed in Table S5. See also Figure S1.

Immunoblotting was performed on the wild-type, *BAX*^*−/−*^, *PTEN*^*−/−*^, and *XIAP*^*−/−*^ HCT116 cell lines using antibodies against AKT, p-AKT Ser^473^, PTEN, XIAP, and BAX (tubulin was used as a loading control) (Figure 5A). AKT levels in the three knockout cell lines were similar to those in wild-type (Figure 5B). The same was true for p-AKT Ser^473^ in the *BAX*^−/−^ and *XIAP*^−/−^ lines, but p-AKT Ser^473^ was significantly higher in the *PTEN*^*−/−*^ line compared with the wild-type (p < 0.05; Figure 5C), as would be expected with no PTEN-imposed AKT inhibition. The PTEN level in the *BAX*^*−/−*^ line did not differ significantly from wild-type, but it was significantly higher in the *XIAP*^*−/−*^ line (p < 0.05; Figure 5D), consistent with its role in stimulating PTEN degradation (Van Themsche et al., 2009). XIAP levels did not differ between the *BAX*^*−/−*^ and *PTEN*^*−/−*^ lines (Figure 5E). Surprisingly, however, BAX was not detected in the *PTEN*^*−/−*^ line (Figure 5F). To further examine this unexpected finding, we first carried out qRT-PCR to measure BAX mRNA levels in the *PTEN*^*−/−*^ line. BAX mRNA was detected at the same level in wild-type and *PTEN*^*−/−*^ cells (Figure 5H), arguing that the absence of BAX protein in *PTEN*^*−/−*^ cells was not due to impaired transcription. Treatment with inhibitors of proteasomal (MG132) and/or lysosomal (bafilomycin A1) degradation did not restore BAX protein levels in *PTEN*^*−/−*^ cells, suggesting that the absence of BAX was not caused by rapid protein degradation (data not shown). Furthermore, immunoprecipitation suggested the absence of any interaction between PTEN and BAX in wild-type HCT116 cells (Figure 5J), arguing that PTEN does not help to stabilize BAX under normal conditions. This was further corroborated by small interfering RNA (siRNA)-mediated silencing of PTEN in wild-type HCT116 cells, as suppression of PTEN expression did not lead to reduced levels of BAX (Figure 5K).

In the light of the observed absence of BAX in the *PTEN*^*−/−*^ HCT116 line, it should subsequently be considered as both PTEN and BAX deficient. Additional modeling was therefore performed to simulate the lack of BAX in the *PTEN*^*−/−*^ background (*BAX/PTEN*^*−/−*^; Figure S1). Rather than generating a cleaved PARP concentration-response curve that matched the *PTEN*^*−/−*^ scenario, this model reported a response curve that was almost identical to *BAX*^*−/−*^ (Figure S1A). The *BAX/PTEN*^*−/−*^ response curves for the majority of other protein species also matched *BAX*^*−/−*^ (data not shown), with the following exceptions: the active AKT curve increased in line with *PTEN*^*−/−*^ but instead of peaking and falling to a lower steady state, it continued rising to an approximately 3-fold higher steady state (Figure S1B); the XIAP curve began following the trajectory of *PTEN*^*−/−*^, but converged with *BAX*^*−/−*^ at the 6-h mark (Figure S1C).

### AKT Regulates Early TRAIL-Induced Apoptosis

**Figure 6:**
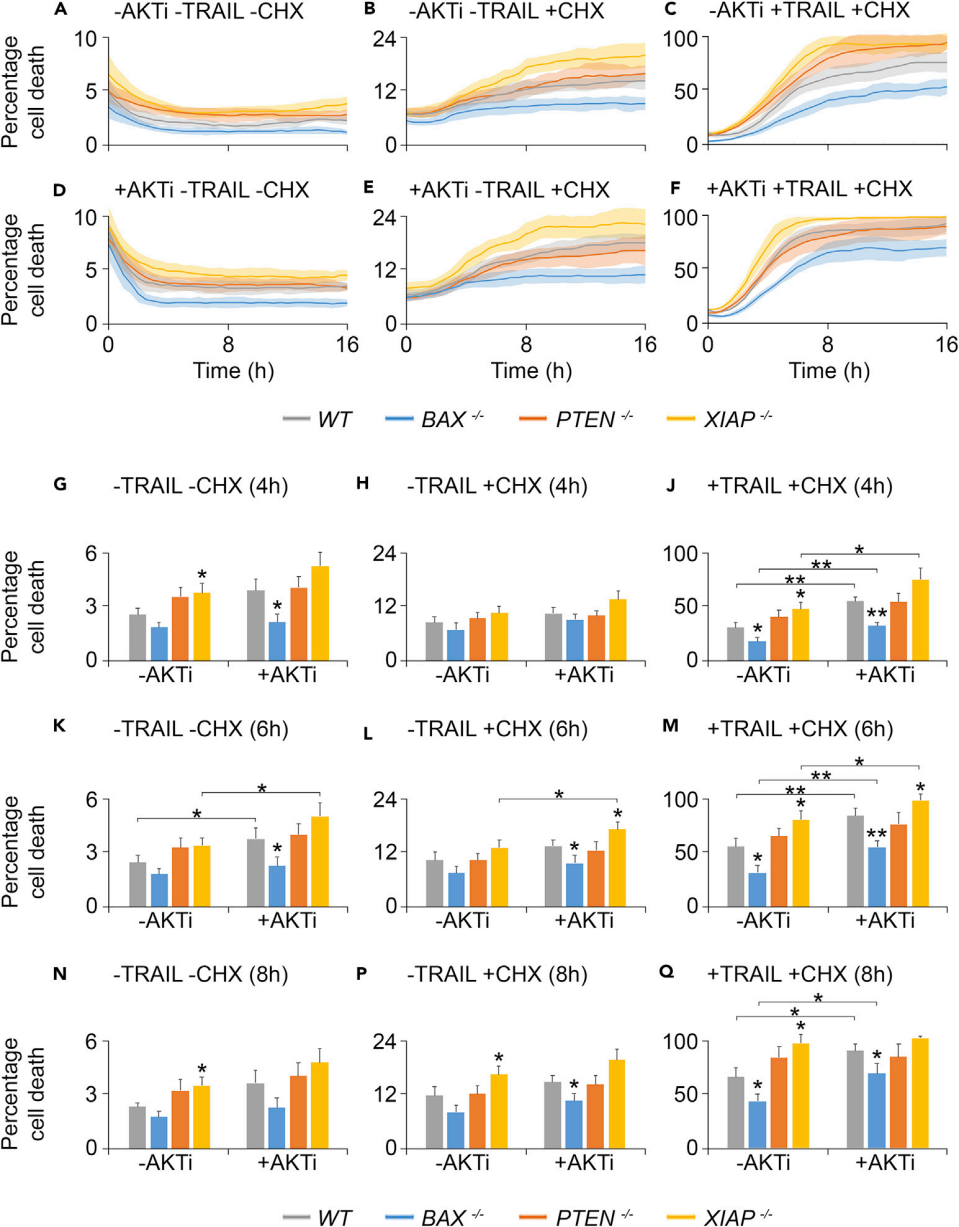
AKT Regulates Early TRAIL-Induced Apoptosis (A–F) IncuCyte-derived percentage cell death in wild-type, *BAX*^*−/−*^, *PTEN*^*−/−*^, and *XIAP*^*−/−*^ HCT116 cell lines over a 16-h time course. +AKTi treatments contained 27.6 μg/mL AKT1/2 kinase inhibitor; +TRAIL treatments, 50 ng/mL TRAIL; and +CHX treatments, 2.5μg/mL cycloheximide. Data in (A–F) represent means ± SD from three independent biological repeats taken at 30-min intervals. SDs are represented by shaded areas. For clarity, y axes (% cell death) are not all scaled to 100%. Treatments: (A) -AKTi -TRAIL –CHX, (B) -AKTi -TRAIL + CHX, (C) -AKTi + TRAIL + CHX, (D) +AKTi -TRAIL –CHX, (E) +AKTi -TRAIL + CHX, and (F) +AKTi + TRAIL + CHX. (G–Q) Time slices of A–F at 4- (G–J), 6- (K–M), and 8-h (N–Q) treatment, showing the effects of AKT inhibition across all TRAIL/CHX treatments. SDs here are represented by error bars. Asterisks above individual bars indicate values significantly different from wild-type within each treatment, whereas asterisks above lines spanning treatments indicate values from the same cell line that differ significantly between treatments. *p ≤ 0.05 (Welch two-sample t test); **p ≤ 0.01. Detailed statistical results are displayed in Table S5. See also Figures S2 and S3.

To complement the modeling data, we next focused on AKT and its temporally variable effects on apoptosis using an IncuCyte imaging system. Wild-type and homozygous knockout HCT116 cells were treated with an AKT1/2 inhibitor (AKTi), in combination with TRAIL and/or CHX (Figure 6: panels A–F show cell death over a 16-h time course; panels G–Q show time-slice data at 4, 6, and 8 h). Immunoblotting provided evidence for increased AKT activity following TRAIL treatment, as measured by increased p-AKT Ser^473^ levels, and demonstrated that the AKTi was effective (Figure S2).

Basal cell death rates were highest in the *XIAP*^*−/−*^ line in the absence or presence of AKTi, and lowest in the *BAX*^*−/−*^ line (Figures 6A, 6D, 6G, 6K, and 6N). Variation from wild-type was recorded for the *XIAP*^*−/−*^ line in the absence of AKTi (significantly higher at 4 and 8 h in the absence of AKTi; Figures 6G and 6N), and for *BAX*^*−/−*^ (significantly lower at 4 and 6 h with AKTi; Figures 6G and 6K). Inclusion of CHX (Figures 6B, 6E, 6H, 6L, and 6P) caused a 4- to 5-fold increase in basal cell death kinetics across all cell lines (relative to untreated cells; compare Figures 6A and 6D with Figures 6B and 6E). The *XIAP*^*−/−*^ line again displayed the most rapid cell death kinetics, and the *BAX*^*−/−*^ line the slowest, although in the absence of AKTi, *XIAP*^*−/−*^ cell death was significantly greater than wild-type only at the 8-h mark (Figure 6P). In the presence of CHX and AKTi, cell death was significantly higher in *XIAP*^*−/−*^ than wild-type cells (6 h; Figure 6L), and lower in *BAX*^*−/−*^ cells (6 and 8 h; Figures 6L and 6P). AKT inhibition in CHX-treated cells altered cell death responses only in the *XIAP*^*−/−*^ line at the 6-h mark (significantly higher; Figure 6L).

Treatments with both CHX and TRAIL (Figures 6C and 6F) generated considerably higher levels of cell death in all cell lines, as anticipated. Cell death levels differed significantly from wild-type across all time points in both the *BAX*^*−/−*^ (lower in the absence and presence of AKTi) and *XIAP*^*−/−*^ (higher in the absence of AKTi). In the *XIAP*^*−/−*^ line in the presence of AKTi, cell death was significantly higher than wild-type only at the 6-h point as cell death approached 100% for both conditions (Figure 6M). Of note, AKT inhibition increased cell death rates most effectively in TRAIL/CHX-treated cells, and this was especially apparent at the 4- and 6-h points (Figures 6J and 6M).

An important finding from these cell death experiments was that cell death in the *PTEN*^*−/−*^ line did not differ significantly from that of wild-type under any condition (despite these cells apparently lacking BAX; Figures 5A and 5F). Furthermore, there were no instances wherein AKT inhibition significantly altered cell death rates in the *PTEN*^*−/−*^ line, arguing that PTEN has a negligible influence over cell death dynamics during TRAIL-induced apoptosis. In support of this, cell death analysis by caspase activity in cells transfected with GFP, wild-type PTEN, or catalytically inactive C124S PTEN (Myers et al., 1997) suggested that overexpression of PTEN in active or inactive forms did not alter apoptosis kinetics in cells treated with TRAIL (Figure S3).

## Discussion

Mathematical modeling can be used to generate predictions of how complex systems, such as apoptosis, behave under different conditions. Here, we have constructed a simplified model to describe the dynamics of the extrinsic apoptotic pathway with sufficient accuracy to provide a framework to test predictions in the laboratory. Using cleaved PARP concentration as an indicator of apoptosis (Figure 2M), the model behaved as predicted for both the removal of BAX and XIAP, with a delayed and hastened onset of apoptosis, respectively. These predictions were based on the same *BAX*^*−/−*^ HCT116 cell line used in this study having previously been used to demonstrate a delayed—albeit not abolished—apoptotic response (Zhang et al., 2000, Wang and Youle, 2012), and the *XIAP*^*−/−*^ HCT116 cell line having demonstrated the opposite (Cummins et al., 2004). The model did not, however, simulate the large expected delay in onset for the removal of PTEN. This expectation was based on the known role of PTEN as a tumor suppressor (Li et al., 1997, Lee et al., 2004). To our knowledge, the *PTEN*^*−/−*^ HCT116 cell line has not previously been used for the purpose of this study.

Using TRAIL as a death-inducing ligand, cell death kinetics very closely resembled the sigmoid-shaped curves of cleaved PARP concentration from the simulation (Figure 2M). More specifically, the *BAX*^*−/−*^ and *XIAP*^*−/−*^ cell lines responded as predicted by the model, with a later onset and earlier onset of cell death, respectively. HCT116 cells are known to be Type II cells (Bentele et al., 2004, Aldridge et al., 2011, Gillissen et al., 2013, Huang et al., 2016), and several studies have suggested that whether a cell follows this behavior or that of Type I depends largely on the relative concentrations of various key apoptotic proteins (Aldridge et al., 2011, Gillissen et al., 2013, Scaffidi et al., 1998). In Type II cells, caspase-8 does not activate sufficient caspase-3 to trigger apoptosis, largely due to the overwhelming concentration of XIAP, meaning that amplification through BAX-mediated MOMP is needed (Scaffidi et al., 1998). Aldridge et al. (2011) showed that the defining factor for Type II cells is a high XIAP to caspase-3 concentration ratio, but that there might also be other factors involved such as the efficiency of caspase-8-mediated BID cleavage (Özören and El-Deiry, 2002). Some studies have also shown that within a single cell line, genetic or pharmacological manipulation affecting the expression of certain proteins—such as XIAP or BAX/BAK—can cause Type II cells to adapt to Type I behavior, and that the extent to which this occurs is concentration dependent rather than being a binary switch between the two (Aldridge et al., 2011, Gillissen et al., 2013).

In the present study, the removal of BAX from the system slowed down the onset of apoptosis, but did not prevent it entirely. This may be in part due to the cells adapting to more of a Type I behavior, and it may also be partly because these cells were only *BAX*^*−/−*^ and not double *BAX/BAK*^*−/−*^ (required to fully disable the mitochondrial apoptotic pathway; Zhang et al., 2000). In the absence of XIAP, the very rapid apoptotic response suggests that these cells are reverting to Type I. Indeed, Gillissen et al. (2013) showed that silencing XIAP led to mitochondria-independent cell death (Type I) in *BAX/BAK*^*−/−*^ HCT116 cells.

The *PTEN*^*−/−*^ line, however, did not match model predictions, having an earlier onset similar to the *XIAP*^*−/−*^ line. This is counterintuitive, as PTEN is an indirect inhibitor of AKT, which itself is an inhibitor of apoptosis. Strikingly, BAX protein was found to be missing in the *PTEN*^*−/−*^ line (Figures 5A and 5F), although surprisingly its transcript was present at control levels. Further analysis of BAX stability in *PTEN*^*−/−*^ cells treated with proteasomal and/or lysosomal inhibitors suggested that the absence of BAX protein was not due to enhanced turnover. In addition, siRNA silencing of PTEN expression in wild-type HCT116 cells was not associated with a marked reduction in BAX, arguing against direct causality between PTEN expression and BAX protein levels. Taken together, our data imply that some other form of post-transcriptional regulation of BAX in these cells is resulting in its absence.

According to both the model and the experimental data, the absence of BAX should cause a substantial delay in apoptotic onset, yet this was not observed in the *PTEN*^*−/−*^ line. Additional modeling of a hypothetical BAX/*PTEN*^*−/−*^ cell line produced a cleaved PARP response curve almost identical to that of the *BAX*^*−/−*^ line (Figure S1), showing that the current model cannot fully explain this anomaly. This additional modeling does, however, suggest that PTEN has some influence on XIAP early in the apoptotic response and on AKT throughout, but this is still not sufficient to significantly affect the overall apoptotic dynamics. In the model, the *BAX/PTEN*^*−/−*^ response curve for cleaved PARP behaves as *BAX*^*−/−*^, suggesting a much stronger influence of BAX than PTEN on the system. As the percentage of cell death in the *PTEN*^*−/−*^ cell line did not significantly differ from that in the wild-type, the absence of BAX in this background is less influential. This could imply XIAP-imposed compensatory regulation on the *PTEN*^*−/−*^ system, because free XIAP levels are likely to be higher in the absence of one of its substrates (PTEN), as the model suggests (Figure 2J). This was not, however, reflected in the total XIAP levels (Figure 5E). Overall, this is indicative of the noise that can often be introduced when attempting to characterize a nonlinear complex system.

Eigenanalysis of the wild-type model revealed AKT, cytochrome *c,* and PARP as the protein species most sensitive to perturbations. The mitochondrial release of cytochrome *c* marks the point of no return in a dying cell and PARP cleavage is the ultimate readout of the model, whereas AKT acts on the system before MOMP and was therefore a good candidate to manipulate experimentally. Investigating AKT dynamics also proved interesting from a modeling perspective because the removal of AKT predicted a large increase in active BAX concentration (Figure 2D) and advanced cytochrome *c* mitochondrial release similar to the *XIAP*^*−/−*^ simulation (Figure 2G), yet minor advancement in the cleavage of PARP (Figure 2M).

In subsequent live-cell imaging experiments, AKT inhibition advanced cell death to the greatest extent during early TRAIL-dependent apoptosis, but did so in all cell lines except *PTEN*^*−/−*^ (Figure 6). In addition, *PTEN*^*−/−*^ cells did not differ significantly from wild-type under any treatment, despite their lax of BAX protein. In addition to the possible XIAP-imposed compensatory effect proposed earlier, the inherent interconnectivity of AKT in numerous cellular signaling pathways might explain this unexpected finding. It is possible that one or more of the myriad interactions between AKT and other signaling pathways are causing compensatory effects in the absence of PTEN, resulting in variability in the very nature of the apoptotic regulatory mechanisms of AKT. Pursuing an answer to this question represents a prime example of how the numerous potential protein interactions inherent to the study of complex systems require an interdisciplinary approach.

### Limitations of the Study

An obvious limitation of any experimental work that uses cell lines in culture is whether a given cell type is representative of its tissue of origin or cells in general. This is particularly true for transformed cells derived from human tumors, for example, that accumulate specific mutations likely to alter their cell growth and/or cell death kinetics. Although in this case our computer-based model has been able to predict with some accuracy the cell death kinetics of a cell line in culture (in this case, HCT116 cells)—arguing that there is a reasonable level of functional plasticity in the mode—it is unlikely that such a predictive system would be applicable to all cells. There is direct evidence for this in our comparisons of predictive and actual cell death kinetics in the *PTEN*^*−/−*^ cell line. With respect to this cell line, as we were unable to identify the precise mechanism behind the absence of BAX protein in *PTEN*^*−/−*^ cells, we must also consider the use of this cell line as a limitation of the study.

## Methods

All methods can be found in the accompanying Transparent Methods supplemental file.

## Data and Software Availibility

Raw data for the cell death assays (Figure 4), Western blot quantification (Figure 5), and AKT inhibition experiments (Figure 6) are available online via a Mendeley Data repository with DOI links as follows: Cell death assays (Figure 4): https://doi.org/10.17632/hvwswmgg7p.1 Western blot quantification (Figure 5): https://doi.org/10.17632/x8v9937psj.1 AKT inhibition experiments (Figure 6): https://doi.org/10.17632/74pf4wwdd4.1.

## Supplemental Information

Document S1. Transparent Methods, Figures S1–S3, and Tables S1–S4

Table S5. Statistical Results, Related to Figures 4–6 Significant results are presented with asterisks (*p ≤ 0.05, **p ≤ 0.01, ***p ≤ 0.001). t values and degrees of freedom (df) are shown to 2 decimal places (d.p.) and p values to 5 d.p. Quantification of immunoblots for Figure 5 were performed after normalizing the wild-type cell line to 1. Where t values are -Inf (negative infinity) with p values of 2.2 ×e^−16^, these are all cases where three independent biological repeats of zero (with therefore zero SD) are tested against the wild-type cell line (equal to 1). One-tailed t tests were used for Figure 5, and Welch two-sample t tests were used in all other cases.

Data S1. MATLAB Code Files, Related to Figures 1–3 ZIP file containing all MATLAB code files required to run and analyze the model. Files include *NEWintegrate_DLE,* runs the model by calling the following files; *NEWinitials,* the model's initial conditions; *NEWrates2,* the model's reaction rate constants; NEWdegrhsVRoff2, the function containing all of the model's ODEs; and *NEWeigenanal,* eigenanalysis file (not called by *NEWintegrate_DLE*).
