# Supplementary material for: Mathematical Modeling Highlights the Complex Role of AKT in TRAIL-Induced Apoptosis of Colorectal Carcinoma Cells
Source: iScience. 2019 Jan 14;12:182–93. doi: 10.1016/j.isci.2019.01.015 (PMC6354781; doi:10.1016/j.isci.2019.01.015)
Supplement: Document S1. Transparent Methods, Figures S1–S3, and Tables S1–S4 [file mmc1.pdf]

ISCI, Volume *12*

Supplemental Information

Mathematical Modeling Highlights the Complex Role of AKT in TRAIL-Induced Apoptosis of Colorectal Carcinoma Cells

Matthew W. Anderson, Joanna J. Moss, Robert Szalai, and Jon D. Lane

Figure S1. Comparisons of protein concentration dynamics as predicted by the AKTM. Related to Figure 5.
Response curves in the wild-type, *BAX*$^{-/-}$, *PTEN*$^{-/-}$, *XIAP*$^{-/-}$, and *BAX/PTEN*$^{-/-}$ modelled cell lines for the following proteins: (A) cleaved PARP; (B) active AKT; (C) XIAP.

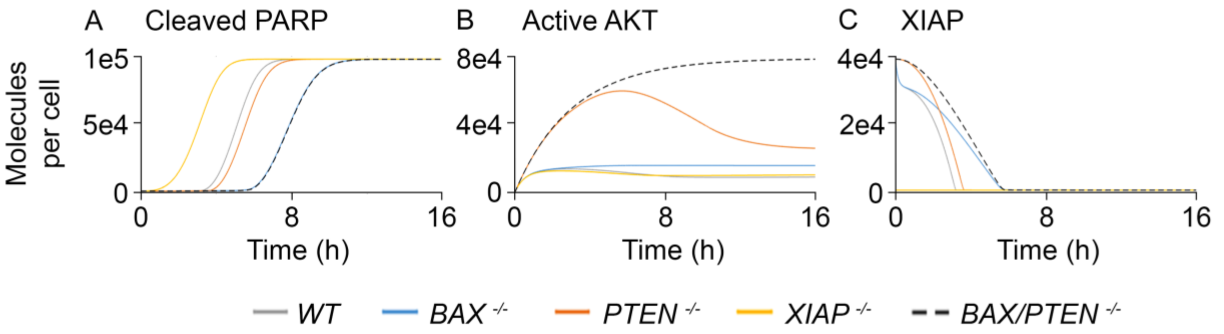

Figure S2. TRAIL treatment causes increased AKT activity. Related to Figure 6.
Immunoblot of wild-type HCT116 cells treated with CHX in the absence or presence of TRAIL, with or without AKTi (as above). Lysates were blotted for total and p-AKT Ser^{473}.

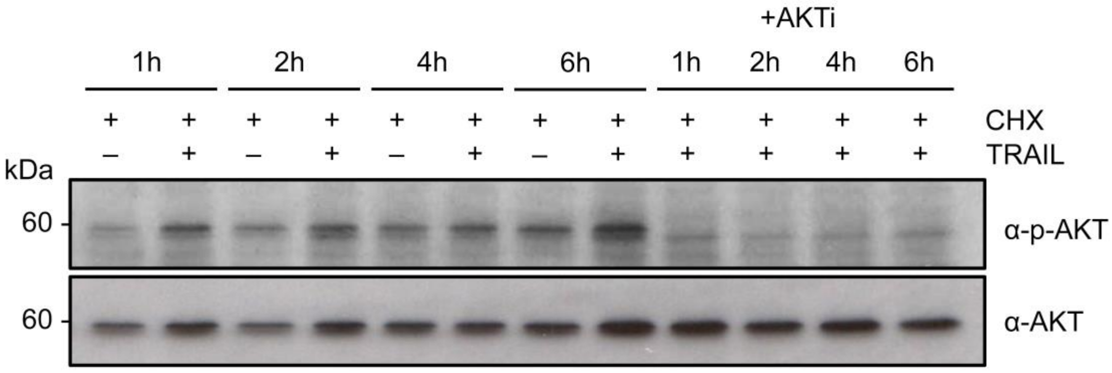

Figure S3. PTEN does not alter apoptosis in cells treated with TRAIL. Related to Figure 6.
Fluorometric caspase assays analyzing cell death in wild-type HCT116 cells transfected with IRES-GFP, PTEN-IRES-GFP or C124S catalytic mutant PTEN-IRES-GFP.

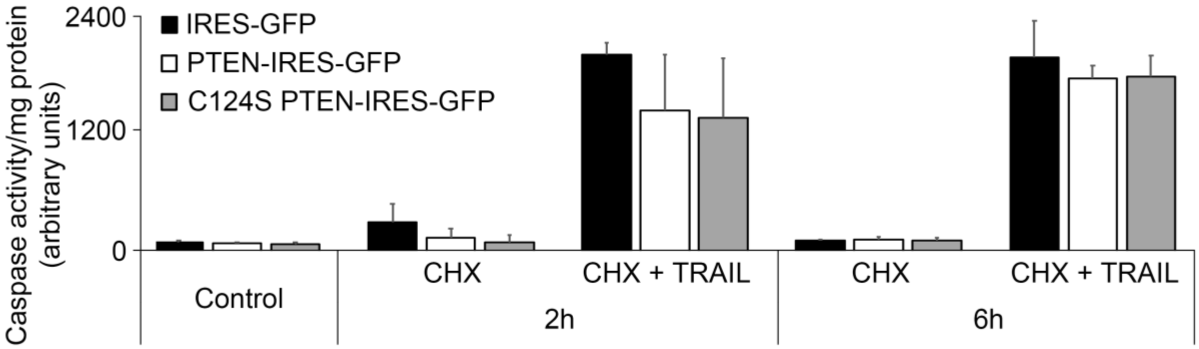

Table S1. List of Protein Species in the AKTM. Related to Figures 1 and 2.
Protein ID corresponds to the xp_i (protein concentration differential), x_i (protein concentration), s_i (synthesis rate constant), and r_i (degradation rate constant) subscripts, *i*, in Table S4.

Protein ID (*i*)	Protein Species	Description
1	TRAIL	TNF-related apoptosis-inducing ligand
2	pC8	Procaspase-8/10; inactive caspase-8/10
3	C8*	Cleavage-activated Caspase-8/10
4	pC3	Procaspase-3/7; inactive caspase-3/7
5	C3*	Cleavage-activated Caspase-3/7
6	BAX	Bcl-2-associated X protein (inactive, cytosolic)
7	BAX*	BAX (active, cytosolic)
8	Mito	Mitochondrial pores; unoccupied BAX_m binding sites
9	Mito*	BAX_m-bound mitochondrial pores (active)
10	$Cytoc_m$	Cytochrome c (mitochondrial)
11	$Cytoc_c$	Cytochrome c (cytosolic)
12	$SMAC_m$	Second mitochondria-derived activator of caspases (mitochondrial)
13	$SMAC_c$	SMAC (cytosolic)
14	PTEN	Phosphatase and tensin homolog
15	AKT	Protein kinase B / AKT
16	AKT*	AKT (active)
17	AKT:$Cytoc_c$	Reaction intermediary
18	Bcl-2	B-cell lymphoma 2
19	Bcl-2:BAX_m	Reaction intermediary
20	XIAP	X-linked inhibitor of apoptosis protein
21	$SMAC_c$:XIAP	Reaction intermediary
22	BAX_m	BAX (active, mitochondrial)
23	AKT:BAX*	Reaction intermediary

24	PARP	Poly(ADP-ribose) polymerase
25	cPARP	Caspase-3/7-cleaved PARP
26	TRAIL:pC8	Reaction intermediary
27	C8*:pC3	Reaction intermediary
28	C3*:pC8	Reaction intermediary
29	C8*:BAX	Reaction intermediary
30	Mito:$Cytoc_m$	Reaction intermediary
31	Mito:$SMAC_m$	Reaction intermediary
32	$Cytoc_c$:pC3	Reaction intermediary
33	PTEN:AKT*	Reaction intermediary
34	$SMAC_c$:XIAP:AKT*	Reaction intermediary
35	C3*:PARP	Reaction intermediary
36	AKT*:XIAP	Reaction intermediary
37	C3*:XIAP	Reaction intermediary
38	$C3_{Ub}$	Ubiquitinated caspase-3/7
39	PTEN:XIAP	Reaction intermediary
40	$PTEN_{Ub}$	Ubiquitinated PTEN
41	XIAP:$Cytoc_c$	Reaction intermediary

Table S2. Initial Conditions and Synthesis/Degradation Rates. Related to Figures 1 and 2.

Protein ID (*i*)	Protein Species	Initial Condition (molecules/cell)	Synthesis Rate (s_i, molecules s^{-1})	Degradation Rate (r_i, s^{-1})
1	TRAIL	1,000	0	5.79×10^{-6}
2	pC8	3,500	2.03×10^{-2}	5.79×10^{-6}
3	C8*	0	0	5.79×10^{-6}
4	pC3	100,000	5.79×10^{-1}	5.79×10^{-6}
5	C3*	0	0	2.89×10^{-5}
6	BAX	80,000	4.63×10^{-1}	5.79×10^{-6}
7	BAX*	0	0	5.79×10^{-6}
8	Mito	50,000	2.89×10^{-1}	5.79×10^{-6}
9	Mito*	0	0	5.79×10^{-6}
10	$Cytoc_m$	500,000	2.89	5.79×10^{-6}
11	$Cytoc_c$	0	0	5.79×10^{-6}
12	$SMAC_m$	50,000	2.89×10^{-1}	5.79×10^{-6}
13	$SMAC_c$	0	0	5.79×10^{-6}
14	PTEN	500,000	2.89	5.79×10^{-6}
15	AKT	100,000	5.79×10^{-1}	5.79×10^{-6}
16	AKT*	0	0	5.79×10^{-6}
17	AKT:$Cytoc_c$	0	0	5.79×10^{-6}
18	Bcl-2	200,000	1.16×10^{-1}	5.79×10^{-6}
19	Bcl-2:BAX_m	0	0	5.79×10^{-6}
20	XIAP	300,000	1.74×10^{-1}	5.79×10^{-6}
21	$SMAC_c$:XIAP	0	0	5.79×10^{-6}
22	BAX_m	0	0	5.79×10^{-6}
23	AKT:BAX*	0	0	5.79×10^{-6}
24	PARP	100,000	5.79×10^{-1}	5.79×10^{-6}

25	cPARP	0	0	5.79×10^{-6}
26	TRAIL:pC8	0	0	5.79×10^{-6}
27	C8*:pC3	0	0	5.79×10^{-6}
28	C3*:pC8	0	0	5.79×10^{-6}
29	C8*:BAX	0	0	5.79×10^{-6}
30	Mito:Cytoc$_m$	0	0	5.79×10^{-6}
31	Mito:SMAC$_m$	0	0	5.79×10^{-6}
32	Cytoc$_c$:pC3	0	0	5.79×10^{-6}
33	PTEN:AKT*	0	0	5.79×10^{-6}
34	SMAC$_c$:XIAP:AKT*	0	0	5.79×10^{-6}
35	C3*:PARP	0	0	5.79×10^{-6}
36	AKT*:XIAP	0	0	5.79×10^{-6}
37	C3*:XIAP	0	0	5.79×10^{-6}
38	C3$_{Ub}$	0	0	5.79×10^{-6}
39	PTEN:XIAP	0	0	5.79×10^{-6}
40	PTEN$_{Ub}$	0	0	5.79×10^{-6}
41	XIAP:Cytoc$_c$	0	0	5.79×10^{-6}
N/A	v (volume correction)	0.07	N/A	N/A

Table S3. Reaction Rate Constants. Related to Figures 1 and 2.

Reaction ID corresponds to the k_j (forward reaction rate constant), kr_j (reverse reaction rate constant), and kc_j (catalytic reaction rate constant) subscripts, *j*, in Table S4.

**Reaction ID (*j*)**	**Reaction**	**k_j ((molecules/cell)$^{-1}$ s^{-1})**	**kr_j (s^{-1})**	**kc_j (s^{-1})**
1	TRAIL + pC8 ↔ TRAIL:pC8 → C8*	10^{-7}	10^{-3}	1
2	C8* + pC3 ↔ C8*:pC3 → C8* + C3*	10^{-6}	10^{-3}	1
3	C3* + pC8 ↔ C3*:pC8 → C3* + C8*	3.00×10^{-8}	10^{-3}	1
4	C8* + BAX ↔ C8*:BAX → C8* + BAX*	10^{-7}	10^{-3}	1
5	BAX_m + Mito ↔ Mito*	10^{-6}	10^{-3}	NA
6	Mito* + $Cytoc_m$ ↔ Mito*:$Cytoc_m$ → Mito* + $Cytoc_c$	2.00×10^{-6}	10^{-3}	0.01/v
7	Mito* + $SMAC_m$ ↔ Mito*:$SMAC_m$ → Mito* + $SMAC_c$	2.00×10^{-6}	10^{-3}	0.01/v
8	Mito* → Mito	10^{-4} (s^{-1})	NA	NA
9	$Cytoc_c$ + pC3 ↔ $Cytoc_c$:pC3 → $Cytoc_c$ + C3*	5.00×10^{-9}	10^{-3}	1
10	PTEN + AKT* ↔ PTEN:AKT* → PTEN + AKT	10^{-8}	2.00×10^{-4}	1
11	AKT* + $Cytoc_c$ ↔ AKT*:$Cytoc_c$	10^{-9}	10^{-3}	NA
12	Bcl-2 + BAX_m ↔ Bcl-2:BAX_m	10^{-6}	10^{-3}	NA
13	$SMAC_c$ + XIAP ↔ $SMAC_c$:XIAP	3.50×10^{-6}	10^{-3}	NA
14	AKT ↔ AKT*	10^{-3} (s^{-1})	10^{-6}	NA
15	AKT* + BAX* ↔ AKT*:BAX*	10^{-6}	10^{-3}	NA
16	UNUSED	NA	NA	NA
17	$SMAC_c$ + XIAP + AKT* ↔ $SMAC_c$:XIAP:AKT* → $SMAC_c$:XIAP + AKT	3.50×10^{-6} ((molecules/cell)$^{-2}$ s^{-1})	10^{-3}	1
18	AKT* + XIAP ↔ AKT*:XIAP → AKT + XIAP	10^{-9}	10^{-3}	1

19	C3* + XIAP ↔ C3*:XIAP → $C3_{Ub}$ + XIAP	7.00 x 10^{-5}	1.67 x 10^{-5}	1.67 x 10^{-4}
20	PTEN + XIAP ↔ PTEN:XIAP → $PTEN_{Ub}$ + XIAP	10^{-8}	1.67 x 10^{-3}	1.67 x 10^{-2}
21	BAX* ↔ BAX_m	0.01 (s^{-1})	0.01/v	NA
22	C3* + PARP ↔ C3*:PARP → C3* + cPARP	10^{-7}	10^{-3}	1
23	XIAP + $Cytoc_c$ ↔ XIAP:$Cytoc_c$	10^{-3}	10^{-3}	NA

Table S4. Ordinary Differential Equations (ODEs). Related to Figures 1 and 2.

Protein ID corresponds to the xp_i (protein concentration differential), x_i (protein concentration), s_i (synthesis rate constant), and r_i (degradation rate constant) subscripts, *i*. Subscripts, *j*, for k_j (forward reaction rate constant), kr_j (reverse reaction rate constant), and kc_j (catalytic reaction rate constant) are based on the Reaction ID shown in Table S3.

Protein ID (*i*)	Protein Species	Ordinary Differential Equation
1	TRAIL	$xp_1 = -k_1x_1x_2 + kr_1x_{26} + s_1 - r_1x_1$
2	pC8	$xp_2 = -k_1x_1x_2 + kr_1x_{26} - k_3x_5x_2 + kr_3x_{28} + s_2 - r_2x_2$
3	C8*	$xp_3 = +kc_1x_{26} - k_2x_3x_4 + kr_2x_{27} + kc_2x_{27} + kc_3x_{28} - k_4x_3x_6 + kr_4x_{29} + kc_4x_{29} - r_3x_3$
4	pC3	$xp_4 = -k_2x_3x_4 + kr_2x_{27} - k_9x_{11}x_4 + kr_9x_{32} + s_4 - r_4x_4$
5	C3*	$xp_5 = +kc_2x_{27} - k_3x_5x_2 + kr_3x_{28} + kc_3x_{28} + kc_9x_{32} - k_{19}x_5x_{20} + kr_{19}x_{37} - k_{22}x_5x_{24} + kr_{22}x_{35} + kc_{22}x_{35} - r_5x_5$
6	BAX	$xp_6 = -k_4x_3x_6 + kr_4x_{29} + s_6 - r_6x_6$
7	BAX*	$xp_7 = +kc_4x_{29} - k_{15}x_{16}x_7 + kr_{15}x_{23} - k_{21}x_7 + kr_{21}x_{22} - r_7x_7$
8	Mito	$xp_8 = -(1/v)^2k_5x_{22}x_8 + kr_5x_9 + k_8x_9 + s_8 - r_8x_8$
9	Mito*	$xp_9 = +(1/v)^2k_5x_{22}x_8 - kr_5x_9 - (1/v)^2k_6x_9x_{10} + kr_6x_{30} + kc_6x_{30} - (1/v)^2k_7x_9x_{12} + kr_7x_{31} + kc_6x_{31} - k_8x_9$
10	$Cytoc_m$	$xp_{10} = -(1/v)^2k_6x_9x_{10} + kr_6x_{30} + s_{10} - r_{10}x_{10}$
11	$Cytoc_c$	$xp_{11} = +kc_6x_{30} - k_9x_{11}x_4 + kr_9x_{32} + kc_9x_{32} - k_{11}x_{16}x_{11} + kr_{11}x_{17} - k_{23}x_{20}x_{11} + kr_{23}x_{41} - r_{11}x_{11}$
12	$SMAC_m$	$xp_{12} = -(1/v)^2k_7x_9x_{12} + kr_7x_{31} + s_{12} - r_{12}x_{12}$
13	$SMAC_c$	$xp_{13} = +kc_7x_{31} - k_{13}x_{13}x_{20} + kr_{13}x_{21} - k_{17}x_{13}x_{20}x_{16} + kr_{17}x_{34} - r_{13}x_{13}$
14	PTEN	$xp_{14} = -k_{10}x_{14}x_{16} + kr_{10}x_{33} + kc_{10}x_{33} - k_{20}x_{14}x_{20} + kr_{20}x_{39} + s_{14} - r_{14}x_{14}$
15	AKT	$xp_{15} = +kc_{10}x_{33} - k_{14}x_{15} + kr_{14}x_{16} + kc_{17}x_{34} + k_{18}x_{36} + s_{15} - r_{15}x_{15}$
16	AKT*	$xp_{16} = -k_{10}x_{14}x_{16} + kr_{10}x_{33} - k_{11}x_{16}x_{11} + kr_{11}x_{17} + k_{14}x_{15} - kr_{14}x_{16} - k_{15}x_{16}x_7 + kr_{15}x_{23} - k_{17}x_{13}x_{20}x_{16} + kr_{17}x_{34} - k_{18}x_{20}x_{16} + kr_{18}x_{36} - r_{16}x_{16}$
17	AKT:$Cytoc_c$	$xp_{17} = +k_{11}x_{16}x_{11} - kr_{11}x_{17} - r_{17}x_{17}$
18	Bcl-2	$xp_{18} = -(1/v)^2k_{12}x_{18}x_{22} + kr_{12}x_{19} + s_{18} - r_{18}x_{18}$
19	Bcl-2:BAX_m	$xp_{19} = +(1/v)^2k_{12}x_{18}x_{22} - kr_{12}x_{19} - r_{19}x_{19}$

20	XIAP	$xp_{20} = -k_{13}x_{13}x_{20} + kr_{13}x_{21} - k_{17}x_{13}x_{20}x_{16} + kr_{17}x_{34} - k_{18}x_{16}x_{20} + kr_{18}x_{36} + kc_{18}x_{36} - k_{19}x_5x_{20} + kr_{19}x_{37} + kc_{19}x_{37} - k_{20}x_{14}x_{20} + kr_{20}x_{39} + kc_{20}x_{39} - k_{23}x_{20}x_{11} + kr_{23}x_{41} + s_{20} - r_{20}x_{20}(1/(x_{16}+1))$
21	SMAC:XIAP	$xp_{21} = +k_{13}x_{13}x_{20} - kr_{13}x_{21} + kc_{17}x_{34} - r_{21}x_{21}$
22	BAX_m	$xp_{22} = -(1/v)^2k_5x_{22}x_8 + kr_5x_9 - (1/v)^2k_{12}x_{18}x_{22} + kr_{12}x_{19} + k_{21}x_7 - kr_{21}x_{22} - r_{22}x_{22}$
23	AKT:BAX*	$xp_{23} = +k_{15}x_{16}x_7 - kr_{15}x_{23} - r_{23}x_{23}$
24	PARP	$xp_{24} = -k_{22}x_5x_{24} + kr_{22}x_{35} + s_{24} - r_{24}x_{24}$
25	cPARP	$xp_{25} = +kc_{22}x_{35} - r_{25}x_{25}$
26	TRAIL:pC8	$xp_{26} = +k_1x_1x_2 - kr_1x_{26} - kc_1x_{26} - r_{26}x_{26}$
27	C8*:pC3	$xp_{27} = +k_2x_3x_4 - kr_2x_{27} - kc_2x_{27} - r_{27}x_{27}$
28	C3*:pC8	$xp_{28} = +k_3x_5x_2 - kr_3x_{28} - kc_3x_{28} - r_{28}x_{28}$
29	C8*:BAX	$xp_{29} = +k_4x_3x_6 - kr_4x_{29} - kc_4x_{29} - r_{29}x_{29}$
30	Mito:$Cytoc_m$	$xp_{30} = +(1/v)^2k_6x_9x_{10} - kr_6x_{30} - kc_6x_{30} - r_{30}x_{30}$
31	Mito:$SMAC_m$	$xp_{31} = +(1/v)^2k_7x_9x_{12} - kr_7x_{31} - kc_6x_{31} - r_{31}x_{31}$
32	$Cytoc_c$:pC3	$xp_{32} = +k_9x_{11}x_4 - kr_9x_{32} - kc_9x_{32} - r_{32}x_{32}$
33	PTEN:AKT*	$xp_{33} = +k_{10}x_{14}x_{16} - kr_{10}x_{33} - kc_{10}x_{33} - r_{33}x_{33}$
34	$SMAC_c$:XIAP:AKT*	$xp_{34} = +k_{17}x_{13}x_{20}x_{16} - kr_{17}x_{34} - kc_{17}x_{34} - r_{34}x_{34}$
35	C3*:PARP	$xp_{35} = +k_{22}x_5x_{24} - kr_{22}x_{35} - kc_{22}x_{35} - r_{35}x_{35}$
36	AKT*:XIAP	$xp_{36} = +k_{18}x_{16}x_{20} - kr_{18}x_{36} - kc_{18}x_{36} - r_{36}x_{36}$
37	C3*:XIAP	$xp_{37} = +k_{19}x_5x_{20} - kr_{19}x_{37} - kc_{19}x_{37} - r_{37}x_{37}$
38	$C3_{Ub}$	$xp_{38} = +kc_{19}x_{37} - r_{38}x_{38}$
39	PTEN:XIAP	$xp_{39} = +k_{20}x_{14}x_{20} - kr_{20}x_{39} - kc_{20}x_{39} - r_{39}x_{39}$
40	$PTEN_{Ub}$	$xp_{40} = +kc_{20}x_{39} - r_{40}x_{40}$
41	XIAP:$Cytoc_c$	$xp_{41} = +k_{23}x_{20}x_{11} - kr_{23}x_{41} - r_{41}x_{41}$

Transparent Methods

Contact for Reagent and Resource Sharing

Further information and requests for resources and reagents should be directed to and will be fulfilled by the Lead Contact, Matthew Anderson (m.w.anderson@exeter.ac.uk).

Experimental Model and Subject Details

Cell Lines

The HCT116 cell line (Cat. No. CCL-247, ATCC; RRID: CVCL_0291) was obtained from ATCC (Manassas, Virginia, USA). *BAX*$^{-/-}$ HCT116, *PTEN*$^{-/-}$ HCT116, and *XIAP*$^{-/-}$ HCT116 cell lines were obtained from Bert Vogelstein (John Hopkins University, Maryland, USA). All cells were stored at -80°C and subsequently grown in high-glucose DMEM medium (Cat. No. D5796, Sigma) prepared with 10% FBS (Cat. No. 10270106, Thermo Fisher Scientific) in an incubator at 37°C with 5% CO_2. These standard growing conditions were used throughout this study prior to the addition of any treatments.

Method Details

Model Structure

The AKT Apoptosis Model (AKTM) was constructed using MATLAB (Mathworks) and, although there are other numerous mathematical models of apoptosis present in the literature to draw from (Bentele *et al.*, 2004; Bertaux *et al*., 2017; Bialik *et al.*, 2010; Lavrik, 2010; Schleich and Lavrik, 2013), ours is based on the Extrinsic Apoptosis Reaction Model (EARM) series (Albeck *et al.*, 2008; Aldridge *et al.*, 2011; Chen *et al.*, 2007; Cui *et al.*, 2008; Howells *et al.*, 2011; Spencer *et al.*, 2009) with Ordinary Differential Equations (ODEs) describing temporal variation in protein concentrations. This was primarily due to some of these EARM models having focused on HCT116 cells. With the exception of parameters relating to AKT, PTEN and their intermediaries, all of which were fitted to preliminary experimental data for this study, all other rate constants and initial conditions were parameterized according to values for HCT116 cells already obtained in Aldridge *et al.* (2011).

Certain simplifications were applied in its construction, including the omission of caspase-8 activation of BID, the formation of the apoptosome, and the involvement of caspase-6 in the positive feedback loop of the caspase cascade. In doing so, the fundamental mechanisms involved in the system have been conserved (see Figures 1A and 1B for comparison).

Table S1 lists the protein species used in the AKTM, Table S2 lists the protein-specific rate constants and initial conditions, and Table S3 lists the reaction-specific rate constants.

The simple rate equation, $r = k[A]^x[B]^y$ is used for bimolecular reactions, where $[A]$ and $[B]$ are the concentrations of protein species A and B, respectively, and x and y are their partial orders of reaction. Since each reaction is modelled as an elementary one, these partial orders are equal to the stochiometric coefficients of each reactant so that in the case of bimolecular reactions, the reaction order is always equal to two. Bimolecular reactions such as that between TRAIL and

procaspase-8 (pC8) are defined as shown in Equation 1

$$TRAIL + pC8 \quad (k_{rev}) \leftrightarrow (k_{for}) \quad TRAIL{:}pC8 \quad \rightarrow (k_{cat}) \quad C8^{*} \qquad \text{(Equation 1)}$$

where the reaction between TRAIL and pC8 progresses according to a forward reaction rate constant, k_{for}, forming a protein-protein intermediary, TRAIL:pC8. This may then either dissociate back into the reactants according to a reverse reaction rate constant, k_{rev}, or form the products through a catalytic reaction rate constant, k_{cat}. These reactions are modelled in terms of protein concentration changes with respect to time, $d[pC8]\ /\ dt$. Each ODE is constructed as shown in Equation 2

$$\frac{d[pC8]}{dt} = -[TRAIL][pC8]k_{for} + [TRAIL{:}pC8]k_{rev} + k_{syn} - [pC8]k_{deg} \qquad \text{(Equation 2)}$$

where k_{syn} and k_{deg} represent the synthesis and degradation rate constants for pC8, respectively. Additional terms following the same format are included if the focal protein species is involved in multiple reactions. All of the ODEs used in this model are detailed in Table S4.

Maintaining consistency with previous EARMs—EARM v1.0 (Albeck et al, 2008) and v1.4 (Aldridge *et al.*, 2011) in particular—this study has modelled the apoptotic system as having separate cytosolic and mitochondrial compartments, the latter being approximately 7% of the former in volume (Posakony *et al.*, 1977). Mitochondrial protein concentrations were, therefore, adjusted according to a scale factor of $1/v$ to reflect this in rate equations, where $v = 0.07$. However, these previous studies had not accounted for bimolecular mitochondrial reactions, where a scale factor of $\left(\frac{1}{v}\right)^2$ is required to scale the concentrations of both protein species involved in the reaction, as in this study.

Model Parameters

Basal protein concentrations for the simulated HeLa cells in EARM v1.0 (Albeck *et al.*, 2008) were originally extracted from the literature, where available (Eissing, 2004; Rehm *et al.*, 2006; Waterhouse *et al.*, 2001). For unknown protein concentrations, these parameters were fitted to data within an observed generalized range of approximately 1–1,000 nM (Berg, 1985). EARM v1.4 (Aldridge *et al.*, 2011), however, modelled the HCT116 cell line (as in this study) and found the basal concentrations of key proteins through quantitative immunoblotting. In this study, we have extracted all of the applicable parameters from EARM v1.4 and fitted the remaining parameters sequentially through trial-and-error to preliminary data obtained for the wild-type HCT116 cell line (as in Figure 4C), keeping these within the 1–1,000 nM observed generalized range.

Protein synthesis rates (k_{syn}) were excluded from the original EARM v1.0 due to the use of cycloheximide (CHX), a eukaryotic protein synthesis inhibitor. However, it was found that CHX used at 2.5 µg/ml in previous studies did not completely inhibit synthesis, but instead reduced it by around 85% (Aldridge *et al.*, 2011; Ceccarini and Eagle, 1976). Synthesis rates vary between proteins and are only used for those present in the system before any reactions take place (i.e. reactants; excluding reaction intermediaries and products) (Table S2). Degradation rates (k_{deg}) were also excluded from EARM v1.0 as they were assumed to be negligible when compared to other reaction rates. We—along with other later models (Aldridge *et al.*, 2011; Spencer *et al.*, 2009)—assumed a half-life of 24 hours with a rate of 5.79 x 10^{-6} s^{-1}, with the exceptions of active caspase-3 (2.89 x 10^{-5} s^{-1}), the active mitochondrial pore (10^{-4} s^{-1}), and ubiquitinated caspase-3 (0 s^{-1}). Active caspase-3 was observed as highly unstable in *XIAP*$^{-/-}$ HCT116 cells (Aldridge *et al.*,

2011), so the five-fold increase in degradation rate was necessary to account for this. Similarly, the BAX multimeric complexes forming the active mitochondrial pores were set to degrade at a much higher rate in order to fit experimental data. Ubiquitinated caspase-3, however, is assumed to be catalytically inactive, thereby acting as a caspase-3 sink in itself with no degradation rate required for the purposes of the model. The synthesis and degradation rates used in our model are listed in Table S2.

Reaction rate constants—listed in Table S3—are based on the diffusion-limited bimolecular reaction rate constants in Albeck et al. (2008) and Aldridge et al. (2011), and any instances where two reactions were simplified and combined into one involved implementing the slower rate constant in the model.

The following model simplifications were performed in the AKTM. For cases where additional reactions not present in previous EARM models were required, default values were initially applied, followed by sequential trial-and-error fitting to data for the wild-type cell line (defaults: k_{for} = 10^{-6} (molecules/cell)$^{-1}$ s^{-1}; k_{rev} = 10^{-3} s^{-1}; k_{cat} = 1 s^{-1}).

1) AKTM reaction: TRAIL + pC8 $\leftrightarrow$ TRAIL:pC8 $\rightarrow$ C8*

Constituent reactions (EARM v1.4):

TRAIL + Receptor $\leftrightarrow$ TRAIL:Receptor $\rightarrow$ DISC

DISC + pC8 $\leftrightarrow$ DISC:pC8 $\rightarrow$ DISC + C8*

2) AKTM reaction: C3* + pC8 $\leftrightarrow$ C3*:pC8 $\rightarrow$ C3* + C8*

Constituent reactions (EARM v1.4):

C3* + pC6 $\leftrightarrow$ C3*:pC6 $\rightarrow$ C3* + C6*

C6* + pC8 $\leftrightarrow$ C6*:pC8 $\rightarrow$ C6* + C8*

3) AKTM reaction: C8* + BAX $\leftrightarrow$ C8*:BAX $\rightarrow$ C8* + BAX*

Constituent reactions (EARM v1.4):

C8* + BID $\leftrightarrow$ C8*:BID $\rightarrow$ C8* + tBID

tBID + BAX $\leftrightarrow$ tBID:BAX $\rightarrow$ tBID + BAX*

4) AKTM reaction: BAX_m + Mito $\leftrightarrow$ Mito*

Constituent reactions (EARM v1.4):

BAX_m + BAX_m $\leftrightarrow$ $BAX2_m$

$BAX2_m$ + $BAX2_m$ $\leftrightarrow$ $BAX4_m$

$BAX4_m$ + Mito $\leftrightarrow$ $BAX4_m$:Mito $\rightarrow$ Mito*

5) AKTM reaction: Mito* + $Cytoc_m$ $\leftrightarrow$ Mito*:$Cytoc_m$ $\rightarrow$ Mito* + $Cytoc_c$

Constituent reactions (EARM v1.4):

Mito* + $Cytoc_m$ $\leftrightarrow$ Mito*:$Cytoc_m$ $\rightarrow$ Mito* + $Cytoc_r$

$Cytoc_r$ $\leftrightarrow$ $Cytoc_c$

6) AKTM reaction: Mito* + $SMAC_m$ $\leftrightarrow$ Mito*:$SMAC_m$ $\rightarrow$ Mito* + $SMAC_c$

Constituent reactions (EARM v1.4):

$Mito^* + SMAC_m \leftrightarrow Mito^*:SMAC_m \rightarrow Mito^* + SMAC_r$

$SMAC_r \leftrightarrow SMAC_c$

7) AKTM reaction: $Cytoc_c + pC3 \leftrightarrow Cytoc_c:pC3 \rightarrow Cytoc_c + C3^*$

Constituent reactions (EARM v1.4):

$Cytoc_c + APAF1 \leftrightarrow Cytoc_c:APAF1 \rightarrow Cytoc_c + APAF1^*$

$APAF1^* + C9 \leftrightarrow Apoptosome$

$Apoptosome + pC3 \leftrightarrow Apoptosome:pC3 \rightarrow Apoptosome + C3^*$

8) AKTM reaction: $XIAP + Cytoc_c \leftrightarrow XIAP:Cytoc_c$

Constituent reactions (EARM v1.4):

$Cytoc_c + APAF1 \leftrightarrow Cytoc_c:APAF1 \rightarrow Cytoc_c + APAF1^*$

$APAF1^* + C9 \leftrightarrow Apoptosome$

$Apoptosome + XIAP \leftrightarrow Apoptosome:XIAP$

9) AKTM reaction: $PTEN + AKT^* \leftrightarrow PTEN:AKT^* \rightarrow PTEN + AKT$

Constituent biochemical reactions:

$PTEN + PIP3 \leftrightarrow PTEN:PIP3 \rightarrow PTEN + PIP2$

$PIP3 + AKT \leftrightarrow PIP3:AKT \rightarrow PIP2 + AKT^*$

10) AKTM reaction: $AKT^* + Cytoc_c \leftrightarrow AKT^*:Cytoc_c$

Constituent biochemical reactions:

$Cytoc_c + APAF1 \leftrightarrow Cytoc_c:APAF1 \rightarrow Cytoc_c + APAF1^*$

$APAF1^* + C9 \leftrightarrow Apoptosome$

$C9 + AKT^* \leftrightarrow C9:AKT^*$

Eigenanalysis

Eigenvalues were calculated and used to quantify how sensitive particular protein species are to small changes in the system. The more positive an eigenvalue's real part—as opposed to the complex number's imaginary part—the more unstable the system is in the direction of the corresponding eigenvector, indicating which protein species will change in concentration the most due to the instability. For example, the index of the smallest absolute value within the eigenvector singles out the protein that will have changed the least at the point of instability, whereas the index of the largest absolute value refers to the protein that is the most sensitive and hence will have changed in concentration the most. This is useful information to have during the modelling process since it can help to simplify the system and thereby focus the model. Moreover, an understanding of which proteins are most sensitive to perturbations allows for more effective targeting of subsequent experimental work and the development of therapeutic drugs. However, eigenvalues are only justified when variables—in this case, protein concentrations—are slow compared to the magnitude of the eigenvalues because they are representations of the instantaneous dynamics occurring around steady states (Fenichel, 1979; Jones, 1995). When the system is near steady state, the time dependence can be thought of as a perturbation and the

magnitude of the eigenvalues indicate the sensitivity to this perturbation. The eigenvectors then indicate how various species are expected to react to this perturbation. The largest relative component of an eigenvalue indicates the protein that is most sensitive, because its variation is expected to be the largest. In the AKTM, identification of the most sensitive proteins was less reliable from 4-6 hours, because the dynamics became relatively fast during this time; a factor that is considered in our analyses.

To calculate eigenvalues, the model simulation was run and the Jacobian matrix calculated at each sampled solution value as a function of time. Mathematically, if x is the system's set of protein concentrations, then $dx/dt = f(x)$, where $f(x)$ is the collection of ODEs describing its dynamics. The Jacobian matrix was then approximated from a small change function (ε) applied to $f(x)$, such that the kth column of the Jacobian matrix as $\varepsilon \rightarrow 0$ is approximately equivalent to $\left(f\left(x + (\varepsilon \times vk)\right) - f(x)\right)/\varepsilon$, where ε is a small perturbation and vk represents the kth unit vector. The eigenvalues of the Jacobian matrix were then computed at each time point and these amassed into a larger matrix of dimensions, $n \times t$, where n is the number of protein species and t is the number of time points. In addition to the eigenvalues, the eigenvectors were also stored in an even larger $n \times n \times t$ matrix. From these two matrices, any protein species represented by positive eigenvalues could be determined, and this information used to infer the protein species most sensitive to perturbations.

Cell Death Assays

In preparation for live-cell microscopy, cells were grown to 70% confluency in 35 mm glass-bottomed dishes (Cat. No. P35G-0-14-C, MatTek Corp.). An Olympus IX-71 inverted microscope (60× Uplan Fluorite objective; 0.65-1.25 NA, oil) was used for imaging, encased by an incubator calibrated to 37°C and 5% CO_2. Three treatments were applied to cells in high-glucose DMEM medium: control (DMEM only); 2.5 µg/ml Cycloheximide (CHX) (Cat. No. C7698, Sigma); and 2.5 µg/ml CHX + 50 ng/ml TNF-related apoptosis-inducing ligand (TRAIL) (SuperKillerTRAIL: Cat. No. ALX-201-115-C010, Enzo Life Sciences). CHX was included to inhibit new protein synthesis and focus on protein interaction dynamics. A concentration of 2.5 µg/ml has been shown to reduce synthesis rates to 15% of their normal values (Aldridge *et al.*, 2011; Ceccarini and Eagle, 1976), a change that is reflected in the synthesis rates of the model. All treatments were applied to the following four cell lines immediately prior to imaging: HCT116; *BAX*$^{-/-}$ HCT116; *PTEN*$^{-/-}$ HCT116; and *XIAP*$^{-/-}$ HCT116. Images were taken with a CoolSNAP HQ CCD camera (Photometrics) at 5-minute intervals for 16 hours and videos created from these using MetaMorph software (Molecular Devices). Further cell death assays were carried out in wild-type HCT116 cells transiently expressing IRES-GFP, PTEN-IRES-GFP or catalytically inactive C124S PTEN-IRES-GFP (provided by Prof. Kate Nobes, Bristol). For these assays, 75,000-100,000 HCT116 cells were reverse transfected in 96-well plates with the above constructs, and 48 hours later, treated with 2.5 µg/ml CHX in the absence or presence of 50ng/ml TRAIL for 2 or 6 hours. Cells were then lysed and assayed for caspase activity using the EnzChek™ Caspase-3 Assay Kit Z-DEVD-AMC substrate (Cat. No. E13183, Thermo Fisher) according to manufacturer's instructions. Cell lysates were transferred to Costar 96-well black clear bottom plates (Cat. No. 10530753, Thermo Fisher), and fluorescence was measured in Glomax plate reader (Promega; 365nm excitation, 410–465 emission).

Immunoblotting

Cells were grown to 80% confluency in 12-well plates (Cat. No. 3513, Corning) and rinsed with 2 ml ice-cold Phosphate Buffered Saline (PBS) (Cat. No. BR0014G, Thermo Fisher Scientific). The cells in each well were lysed with 100 µl RIPA buffer (Cat. No. 89900, Thermo Fisher Scientific) and lysates kept on ice with regular agitation for 15 minutes before being centrifuged at 15,294 *g* for 5 minutes. Supernatants were finally collected and either used directly for protein quantification or stored at -80°C.

Lysate protein quantification was achieved using the standard test tube protocol of the Pierce™ Bicinchoninic Acid (BCA) Protein Assay Kit (Cat. No. 23225, Thermo Fisher Scientific). In brief, the assay was performed by calculating protein concentrations from measured absorbances at 562 nm of BCA/copper complex-reacted protein lysate samples against those of a set of pre-diluted bovine serum albumin (BSA) protein assay standards.

Protein separation was achieved by Sodium Dodecyl Sulfate Polyacrylamide Gel Electrophoresis (SDS-PAGE). 5-20 µl of sample was added to each well so that 15 µg of protein was present in each. Samples were measured against 4 µl Precision Plus Protein™ All Blue Prestained Protein Standard (Cat. No. 1610373, Bio-Rad). Proteins were transferred to nitrocellulose membranes (Cat. No. 11998905, Fisher Scientific UK) using a Trans-Blot® Turbo™ Transfer Kit (Cat. No. 1704270, Bio-Rad). These were blocked either using 5% (w/v) skimmed milk powder or with 5 % BSA in Tris-buffered saline containing 0.1% Triton X-100 (according to the manufacturer's recommendations) and probed with primary antibodies followed by horseradish peroxidase (HRP)-tagged secondary antibodies. Proteins were imaged by enhanced chemiluminescence. Antibodies used are listed in the Key Resources Table.

qRT-PCR

Target cells were grown to confluency in 6-well plates and rinsed with 2 ml ice-cold PBS. RNA was extracted using the RNeasy Mini Kit (Cat. No. 74104, Qiagen), following the manufacturer's instructions. Total RNA was converted into cDNA, following the Life Technologies RNA-to-cDNA protocol and amplified by real-time polymerase chain reaction; 37°C for 60 minutes and 95°C for 5 minutes.

For qRT-PCR, each condition had three distinct triplicates, and each of these run in triplicate to assess pipetting accuracy between samples. Per sample, 2 µl cDNA was added to 0.5 µl of the forward and reverse primers at 5 µM each, 5 µl SYBR-Green (Cat. No. 4309155, Life Technologies) and 2 µl H_2O, to one well of a 96-well plate. GAPDH was used as a protein control for every plate.

Immunoprecipitation

Cells plated on 10 cm dishes were washed with ice-cold PBS and lysed in IP lysis buffer (50 mM Tris HCl; pH 7.5, 150 mM NaCl, 0.5% Triton X-100 and proteinase inhibitor tablet (Cat. No. 11836153001, Sigma)). Samples were incubated on ice for 10 minutes then centrifuged at 13,000 x*g* for 15 minutes at 4°C and the supernatants collected as soluble protein fractions. 200 µg protein was incubated for 3 hours at 4°C with 3 µg of antibody (anti-BAX, anti-PTEN or anti-FLAG as a control (Cat. No. A00187, Genscript)). Samples were then incubated with protein G sepharose beads (Cat. No. ab193259, Abcam) for precipitation for 2 hours at 4°C. Beads were washed four times with IP lysis buffer containing 0.1% Triton X-100 and results were analysed by

immunoblotting, as described above.

siRNA of PTEN in the WT line

Knockdown (KD) experiments were performed using 20 µM siRNA construct to target GL2 (control, Eurofins Genomic: CGUACGCGGAAUACUUCGAUU(dTdT)) or PTEN (Mission® esiRNA; Cat. No. EHU106441, Sigma) using Lipofectamine 2000 (Cat. No. 11668019, Thermo Fisher Scientific) as per the manufacturers' instructions. Cells were left for 48 hours before being lysed as above.

AKT Inhibition Assays

The IncuCyte® imaging system was used to conduct large live-cell experiments with numerous treatments. Through comparisons with previously performed cell death assays, this system was found to be the optimum means of imaging dying cells since any background cell death inherent to confocal imaging and human interference was minimized by the incorporation of an imaging system within the incubator. Target cells were first plated on 96-well plates and grown to 70% confluency. Treatments were applied to wells immediately prior to imaging using a multichannel pipette. Six treatments were applied to all four HCT116 cell lines in fresh high-glucose DMEM: control (DMEM only); 27.6 µg/ml AKT1/2 Kinase Inhibitor (AKTi) (Cat. No. A6730, Sigma); 2.5 µ g/ml CHX; 2.5 µ g/ml CHX + 27.6 µg/ml AKTi; 50 ng/ml TRAIL + 2.5 µg/ml CHX; and 50 ng/ml TRAIL + 2.5 µ g/ml CHX + 27.6 µg/ml AKTi. Manufacturer specifications (Sigma) for AKTi showed an IC50 of 58 nM, 210 nM, and 2.12 mM for AKT1, AKT2, and AKT3, respectively, and a "high concentration" of 50 µM (27.6 µg/ml). 250 nM IncuCyte® Cytotox Red Reagent (Essen BioScience) was used to detect dead cells in the red channel. This reagent fluoresces upon binding to DNA once plasma membrane integrity has been compromised in dying cells. Two images were taken at each well at 30-minute intervals in both the phase contrast and red channels. IncuCyte ZOOM® software (Essen BioScience) was used to process and create videos from these images.

Quantification and Statistical Analysis

All statistical tests were performed in R (https://www.r-project.org/) using the RStudio user interface (https://www.rstudio.com/). Three independent experiments (n = 3) were performed in all cases, from which means and their standard deviations (SDs) were calculated. Statistical significance was defined by a result giving a p-value ≤ 0.05. One-tailed t-tests were used for immunoblots in Figure 5, but all other statistical tests (Figures 4 and 6) were Welch two-sample t-tests. Detailed statistical results for all applicable figures are displayed in Table S5.

Protein Quantification

Immunoblot images were imported into MATLAB and normalized for background brightness. Pixels were then thresholded and counted, from which their mean "greyness" calculated and quantified values produced. Values for all mutant cell lines corresponding to a single antibody were then normalized relative to that of wild-type, and mutant cell line means and their SDs calculated from three separate experiments (n = 3). One-tailed t-tests were then used to test whether the means for each antibody-specific mutant cell line differed significantly from one (the

normalized value for wild-type). In cases where no protein was detected in a cell line—for example, when blotting for BAX in the *BAX*$^{-/-}$ line—statistical testing found a highly significant difference between its mean of zero (with SD = 0) and that of the wild-type cell line (each repeat normalized to 1, therefore also with SD = 0). In these cases, as a consequence of testing zero against one, t-values were negative infinity (-Inf), degrees of freedom (df) were 2, and p-values < 2.2×10^{-16}.

AKT Inhibition Assays

Videos produced in AKT inhibition experiments were analyzed using custom MATLAB code developed using the Miji framework (Daniel Sage, Biomedical Image Group) to incorporate Fiji (Schindelin *et al.*, 2012)—an image analysis suite of ImageJ (https://imagej.nih.gov/ij/)—into the MATLAB environment. This enabled Fiji tools to be utilized through MATLAB, thereby allowing for automated processing and cell counting of numerous large video files. With the exception of an adjustment in thresholding, the process for counting live and dead cells was identical, both involving the application of a bandpass filter followed by finding the local maxima based on the filters used. Each experiment was performed in triplicate (n = 3), from which means and their SDs were calculated at each 30-minute interval. Data were extracted from these experiments from the 4-, 6- and 8-hour time points, and Welch two-sample t-tests performed both between different cell lines within each treatment and between different treatments for each cell line.

Data and Software Availability

Raw data for the cell death assays (Figure 4), Western blot quantification (Figure 5), and AKT inhibition experiments (Figure 6) are available online via a Mendeley Data repository with DOI links as follows:

Cell death assays (Figure 4): http://dx.doi.org/10.17632/hvwswmgg7p.1
Western blot quantification (Figure 5): http://dx.doi.org/10.17632/x8v9937psj.1
AKT inhibition experiments (Figure 6): http://dx.doi.org/10.17632/74pf4wwdd4.1

Key Resources Table

REAGENT or RESOURCE	SOURCE	IDENTIFIER
**Antibodies**		
Anti-AKT (pan) (mouse monoclonal)	Cell Signaling	Cat. No. 2920
Anti-p-AKT S473 (rabbit monoclonal)	Cell Signaling	Cat. No. 4060
Anti-BAX (rabbit polyclonal)	Cell Signaling	Cat. No. 2772
Anti-PTEN (rabbit monoclonal)	Cell Signaling	Cat. No. 9559
Anti-XIAP (rabbit monoclonal)	Cell Signaling	Cat. No. 14334
Anti-α-Tubulin (mouse monoclonal)	Sigma	Cat. No. T5168
Anti-Mouse HRP-conjugated secondary antibody (goat polyclonal)	SignalChem	Cat. No. G32-62G-1000

Anti-Rabbit HRP-conjugated secondary antibody (goat polyclonal)	SignalChem	Cat. No. G33-62G-1000
Anti-GAPDH (mouse monoclonal)	Sigma	Cat. No. G8796
Anti-FLAG (mouse monoclonal)	Genscript	Cat No. A00187
**Chemicals, Peptides, and Recombinant Proteins**		
RIPA buffer	Thermo Fisher Scientific	Cat. No. 89900
Sodium Dodecyl Sulfate (SDS)	Fisher Scientific UK	Cat. No. S520053
30% acrylamide / 0.8% (w/v) bisacrylamide	Severn Biotech	Cat. No. 20-2100-10
Ammonium persulfate (APS)	Bio-Rad	Cat. No. 1610700
Tetramethylethylenediamine (TEMED)	Bio-Rad	Cat. No. 1610800
Precision Plus Protein All Blue Prestained Protein Standards	Bio-Rad	Cat. No. 1610373
ECL Western Blotting Detection Reagents	Sigma	Cat. No. GERPN2209
SuperKillerTRAIL (human)	Enzo Life Sciences	Cat. No. ALX-201-115-C010
Cycloheximide	Sigma	Cat. No. C7698
IncuCyte Cytotox Red Reagent	Essen BioScience	Cat. No. 4632
AKT1/2 Kinase Inhibitor	Sigma	Cat. No. A6730
SYBR-Green	Life Technologies	Cat No. 4309155
Lipofectamine 2000	Thermo Fisher Scientific	Cat No. 11668019
Proteinase inhibitor tablet	Sigma	Cat No. 11836153001
Protein G Sepharose beads	Abcam	Cat No. ab193259
**Critical Commercial Assays**		
Pierce BCA Protein Assay Kit	Thermo Fisher Scientific	Cat. No. 23225
Trans-Blot Turbo Transfer Kit	Bio-Rad	Cat. No. 1704270
RNeasy Mini Kit	Qiagen	Cat No. 74104
**Deposited Data**		
Cell death assays raw data	Mendeley Data	http://dx.doi.org/10.17632/hvwswmgg7p.1
Densitometry data	Mendeley Data	http://dx.doi.org/10.17632/x8v9937psj.1
AKT inhibition assays raw data	Mendeley Data	http://dx.doi.org/10.17632/74pf4wwdd4.1
**Experimental Models: Cell Lines**		
HCT116 (human colorectal carcinoma)	ATCC	Cat. No. CCL-247; RRID: CVCL_0291
HCT116 *BAX*$^{-/-}$	Zhang *et al.* (2000)	N/A
HCT116 *PTEN*$^{-/-}$	Lee *et al.* (2004)	N/A
HCT116 *XIAP*$^{-/-}$	Cummins *et al.* (2004)	N/A
**Software and Algorithms**		

MATLAB	Mathworks	N/A
R	N/A	https://www.r-project.org/
RStudio	N/A	https://www.rstudio.com/
ImageJ	N/A	https://imagej.nih.gov/ij/
Fiji	Schindelin *et al.* (2012)	https://fiji.sc/
Miji	Daniel Sage, EPFL, Switzerland	https://imagej.net/Miji/
IncuCyte ZOOM	Essen BioScience	N/A
MetaMorph	Molecular Devices	N/A
Excel	Microsoft	N/A
AKT Apoptosis Model	This study	N/A
Cell death quantification algorithms	This study	N/A
Protein quantification algorithms	This study	N/A
**Other**		
Dulbecco's Modified Eagle's Medium (DMEM)	Sigma	Cat. No. D5796
Fetal Bovine Serum (FBS)	Thermo Fisher Scientific	Cat. No. 10270106
Phosphate Buffered Saline (PBS)	Thermo Fisher Scientific	Cat. No. BR0014G
0.45 µm nitrocellulose transfer membranes	Fisher Scientific UK	Cat. No. 11998905
Hyperfilm	Sigma	Cat. No. GE28-9068-36
**Primers for qRT-PCR**		
**Gene**	**Forward Primer**	**Reverse Primer**
BAX	5'-CCGCCGTGGACACAGAC-3′	5′-CAGAAAACATGTCAGCTGCCA-3′
GAPDH	5'-TTGAGGTCAATGAAGGGGTC-3'	5'-GAAGGTGAAGGTCGGAGTCA-3'

Supplemental References

Albeck, J.G., Burke, J.M., Spencer, S.L., Lauffenburger, D.A., and Sorger, P.K. (2008). Modeling a snap-action, variable-delay switch controlling extrinsic cell death. PLoS Biol., 6(12), e299.

Berg, O.G. (1985). Orientation constraints in diffusion-limited macromolecular association. The role of surface diffusion as a rate-enhancing mechanism. Biophys. J., 47(1), 1-14.

Bertaux, F., Drasdo, D., and Batt, G. (2017). System modeling of receptor-induced apoptosis. In TRAIL, Fas Ligand, TNF and TLR3 in Cancer, O. Micheau, ed. (Springer), pp. 291-307.

Bialik, S., Zalckvar, E., Ber, Y., Rubinstein, A.D., and Kimchi, A. (2010). Systems biology analysis of programmed cell death. Trends Biochem. Sci., 35(10), 556-564.

Ceccarini, C., and Eagle, H. (1976). Some paradoxical effects of inhibitors of protein synthesis on protein turnover in cultured human cells. In Vitro, 12(5), 346-351.

Chen, C., Cui, J., Lu, H., Wang, R., Zhang, S., and Shen, P. (2007). Modeling of the role of a Bax-activation switch in the mitochondrial apoptosis decision. Biophys. J., 92(12), 4304- 4315.

Cui, J., Chen, C., Lu, H., Sun, T., and Shen, P. (2008). Two independent positive feedbacks and bistability in the Bcl-2 apoptotic switch. PLoS One, 3(1), e1469.

Eissing, T., Conzelmann, H., Gilles, E.D., Allgöwer, F., Bullinger, E., and Scheurich, P. (2004). Bistability analyses of a caspase activation model for receptor-induced apoptosis. J. Biol. Chem., 279(35), 36892-36897.

Fenichel, N. (1979). Geometric singular perturbation theory for ordinary differential equations. J. Differ. Equ., 31(1), 53-98.

Howells, C.C., Baumann, W.T., Samuels, D.C., and Finkielstein, C.V. (2011). The Bcl-2-associated death promoter (BAD) lowers the threshold at which the Bcl-2-interacting domain death agonist (BID) triggers mitochondria disintegration. J. Theor. Biol., 271(1), 114-123.

Jones, C. K. (1995). Geometric singular perturbation theory. In Dynamical Systems, R. Johnson, ed. (Springer Berlin Heidelberg), pp. 44-118.

Lavrik, I.N. (2010). Systems biology of apoptosis signaling networks. Curr. Opin. Biotechnol., 21(4), 551-555.

Posakony, J.W., England, J.M., and Attardi, G. (1977). Mitochondrial growth and division during the cell cycle in HeLa cells. J. Cell Biol., 74(2), 468-491.

Rehm, M., Huber, H.J., Dussmann, H., and Prehn, J.H. (2006). Systems analysis of effector caspase activation and its control by X-linked inhibitor of apoptosis protein. EMBO J., 25(18), 4338-4349.

Schindelin, J., Arganda-Carreras, I., Frise, E., Kaynig, V., Longair, M., Pietzsch, T., Preibisch, S., Rueden, C., Saalfeld, S., Schmid, B., and Tinevez, J.Y. (2012). Fiji: an open-source platform for biological-image analysis. Nat. Med., 9(7), 676-682.

Schleich, K., and Lavrik, I.N. (2013). Mathematical modeling of apoptosis. Cell. Commun. Signal., 11(1), 44.

Spencer, S.L., Gaudet, S., Albeck, J.G., Burke, J.M., and Sorger, P. K. (2009). Non-genetic origins of cell-to-cell variability in TRAIL-induced apoptosis. Nature, 459(7245), 428-432.

Waterhouse, N.J., Goldstein, J.C., Von Ahsen, O., Schuler, M., Newmeyer, D.D., and Green, D.R. (2001). Cytochrome c maintains mitochondrial transmembrane potential and ATP generation after outer mitochondrial membrane permeabilization during the apoptotic process. J. Cell Biol., 153(2), 319-328.
